# TAILoR (TelmisArtan and InsuLin Resistance in Human Immunodeficiency Virus [HIV]): An Adaptive-design, Dose-ranging Phase IIb Randomized Trial of Telmisartan for the Reduction of Insulin Resistance in HIV-positive Individuals on Combination Antiretroviral Therapy

**DOI:** 10.1093/cid/ciz589

**Published:** 2019-07-03

**Authors:** Sudeep Pushpakom, Ruwanthi Kolamunnage-Dona, Claire Taylor, Terry Foster, Cath Spowart, Marta García-Fiñana, Graham J Kemp, Thomas Jaki, Saye Khoo, Paula Williamson, Munir Pirmohamed, for the TAILoR Study Group, Jonathan Ainsworth, David Chadwick, Mas Chaponda, Mayur Chauhan, Duncan Churchill, Satyajit Das, Mark Gompels, Elbushra Hereika, Margaret Johnson, Clifford Leen, David Loay, Fabiola Martin, Jane Minton, Barry Peters, Frank Post, Gabriel Schembri, Jiten Vora, John Whitehead

**Affiliations:** 1 Department of Molecular and Clinical Pharmacology, University of Liverpool, United Kingdom; 2 Department of Biostatistics, University of Liverpool, United Kingdom; 3 Clinical Trials Research Centre, University of Liverpool, United Kingdom; 4 Liverpool Magnetic Resonance Imaging Centre, University of Liverpool, United Kingdom; 5 Department of Mathematics and Statistics, Lancaster University, United Kingdom

**Keywords:** HIV, antiretroviral drugs, insulin resistance, metabolic disease, telmisartan

## Abstract

**Background:**

Combination antiretroviral therapy results in metabolic abnormalities which increase cardiovascular disease risk. We evaluated whether telmisartan reduces insulin resistance in human immunodeficiency virus (HIV)–positive individuals on antiretrovirals.

**Methods:**

We conducted a multicenter, randomized, open-label, dose-ranging controlled trial of telmisartan. Participants with HIV infection receiving combination antiretroviral therapy were randomized equally to either no intervention (control) or 20, 40, or 80 mg telmisartan once daily. The adaptive design allowed testing of all dose(s) of telmisartan in stage I, with the promising dose(s) being taken into stage II. The primary outcome measure was reduction in homeostasis model assessment of insulin resistance (HOMA-IR) at 24 weeks.

**Results:**

A total of 377 patients were recruited. In stage I, 48, 49, 47, and 45 patients were randomized to control and 20, 40, and 80 mg telmisartan, respectively (total n = 189). At the interim analysis, 80 mg telmisartan was taken forward into stage II. At the end of stage II (n = 105, control; 106, 80-mg arm), there were no differences in HOMA-IR (estimated effect, 0.007; SE, 0.106) at 24 weeks between the telmisartan (80 mg) and nonintervention arms. Longitudinal analysis over 48 weeks showed no change in HOMA-IR, lipid or adipokine levels. There were significant (*P* ≤ .05), but marginal, improvements in revised Quantitative Insulin Sensitivity Check Index (QUICKI) (0.004) and plasma hs-CRP (−0.222 mg/L) and reduction in liver fat content (1.714 mean reduction; *P* = .005).

**Conclusions:**

No significant effect of telmisartan was demonstrated on the primary outcome (HOMA-IR), but there were marginal improvements with some secondary outcome measures. Further studies in this population are warranted to identify novel strategies for preventing cardiovascular morbidity and mortality.

**Clinical Trial Registration:**

ISRCTN registry (51069819).

## Teaser

The efficacy of telmisartan in improving insulin resistance in patients with human immunodeficiency virus was investigated in an adaptive-design trial. Telmisartan had no significant effect on HOMA-IR, a measure of insulin resistance, but there were improvements with some secondary outcome measures.

Combination antiretroviral therapy (cART) increases the risk of insulin resistance, obesity, and type 2 diabetes (T2D), predisposing factors for cardiovascular disease (CVD), in human immunodeficiency virus (HIV)–positive individuals [1, 2]. A meta-analysis of 65 studies (n = 55 094) found the prevalence of metabolic syndrome (MS) in HIV-infected individuals to be between 16.7% and 18% [3]. The HIV D:A:D (Data collection on Adverse events of anti-HIV Drugs) cohort (n = 33 347) [4] has shown that the prevalence of MS increases from 19.4% to 41.6% over a 6-year period, with a 4-fold increase in the incidence of T2D and a 2- to 3-fold increased risk of CVD [5]. The increase in CVD risk is seen with both individual antiretroviral (ARV) drugs, including protease inhibitors (PIs) [6] and nucleoside reverse transcriptase inhibitors (NRTIs) [7], and ARV drug combinations [8].

Insulin resistance is central to the development of cardiometabolic disease [9]. In vitro studies [10] and single-drug studies in healthy individuals [11] and HIV-positive patients [12, 13] have shown that both PIs and NRTIs cause insulin resistance. The prevalence of insulin resistance in HIV is ~21% [14] and is seen even with some of the newer ARV drugs [15]. The change in insulin resistance, assessed using the validated surrogate marker, the HOMA-IR, has been shown to occur within 4 weeks of starting therapy [15]. Clinical intervention to arrest or reverse cART-associated insulin resistance may be a strategy to reduce the incidence of T2D and CVD in HIV-positive individuals. To this end, insulin sensitizers such as thiazolidinediones and metformin have been trialed. However, the results from randomized clinical trials (RCTs) in HIV-positive individuals have been mixed [16, 17], and there are concerns about the safety profiles of these agents [18, 19].

Telmisartan, an angiotensin receptor blocker (ARB), reduces insulin resistance in non-HIV patients with T2D or MS [20, 21]. In the The Ongoing Telmisartan Alone and in Combination with Ramipril Global Endpoint Trial (120 000 patient-years of follow-up), it also reduced cardiovascular events in a broad group of at-risk patients and conferred cardiovascular protection similar to ramipril but was better tolerated [22]. A meta-analysis of 33 RCTs where telmisartan was compared against other antihypertensive drugs found telmisartan to significantly reduce insulin resistance [23]. However, this was not a universal finding with some trials of telmisartan showing a reduction in insulin resistance [20, 21, 24, 25].

Adipose tissue, a major mediator of glucose and lipid homeostasis, accumulates ARVs [26]. We [27] and others [28] have shown that telmisartan partially reversed ARV-induced adipocyte toxicity in vitro. This was hypothesized to be due to telmisartan acting as a partial agonist at the adipocyte nuclear receptor peroxisome proliferator–activated receptor γ (PPARγ) [29]. A pilot study in 35 HIV-positive individuals showed that telmisartan led to a small but significant loss in adipose tissue after 24 weeks [30]. However, RCT evidence to show telmisartan has a beneficial effect on insulin resistance in HIV-positive individuals is lacking. In order to fill this evidence gap, we conducted a novel, adaptive-design, phase 2 RCT to assess the efficacy and safety of telmisartan in reducing insulin resistance in cART-treated patients with HIV, as well as identifying the optimal telmisartan dose.

## METHODS

### Aims and Study Design

**Figure 1.**
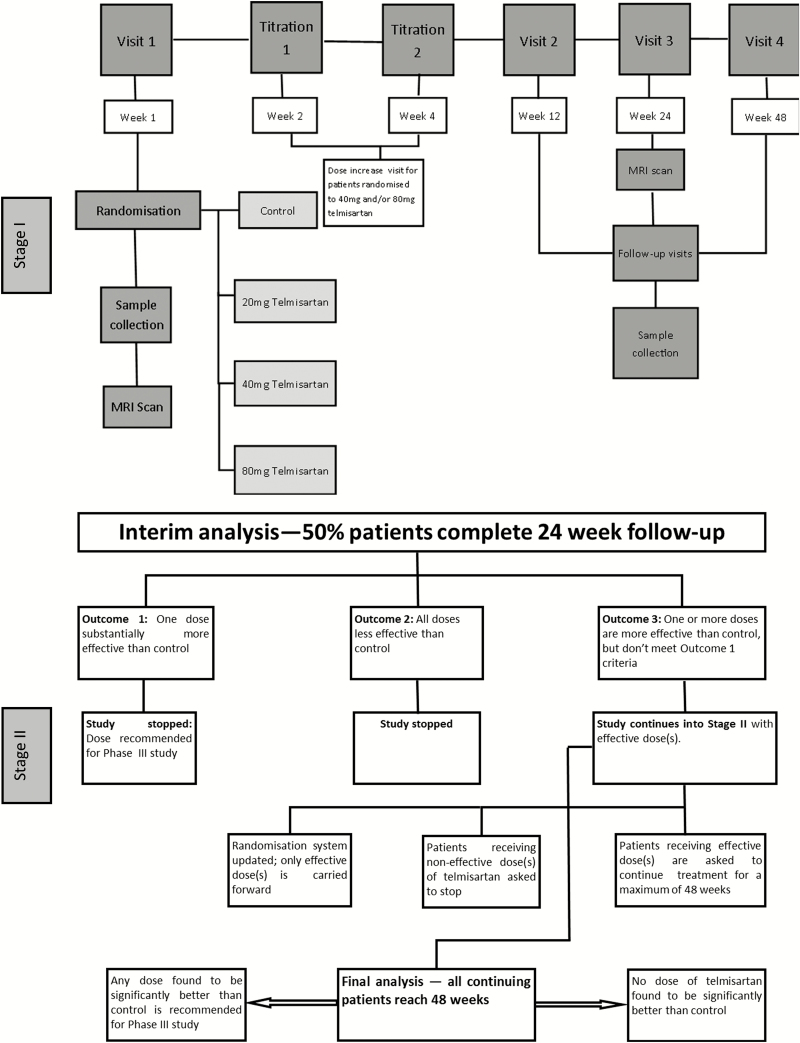
Schematic of the trial design showing 2 different stages of the trial. Stage 1 shows the adaptive-design stage of the trial where participants were randomized equally to no intervention or to 3 different doses of telmisartan (20, 40, and 80 mg). An interim analysis was performed when half of the planned maximum number of patients had been followed up for at least 24 weeks. Stage II shows the 3 different possibilities for the continuation into stage II following the interim analysis. Abbreviation: MRI, magnetic resonance imaging.

TelmisArtan and InsuLin Resistance in HIV (TAILoR) was a multicenter, randomized, open-label controlled trial with an adaptive design (Figure 1) with the aim of determining whether telmisartan reduces insulin resistance in HIV-positive individuals on cART (ISRCTN number 51069819). The trial design, inclusion/exclusion criteria, trial methods, and outcomes have been described previously [31]. The trial (see Supplementary data for the full protocol) was approved by the North West (Liverpool Central) ethics committee. Oversight was provided by a trials steering committee and an independent data and safety monitoring committee.

The adaptive design consisted of 2 stages. Briefly, eligible patients were randomized equally to either no intervention (control, arm A) or 20 mg (arm B), 40 mg (arm C), or 80 mg (arm D) of telmisartan once daily in stage I. This was followed by an interim analysis when half of the planned maximum number of patients had been followed up for at least 24 weeks. Based on the results of interim analysis, there were 3 different possibilities for stage II: (1) if 1 active-dose group was substantially more effective than the control, then the study would have been immediately stopped and the corresponding dose would be taken directly into phase 3; (2) if any active-dose groups showed insufficient promise at the interim analysis, they would be dropped and the study continued with the remaining doses and control for a further 24 weeks; or (3) if no dose showed sufficient promise at the interim analysis, the study would be stopped altogether. The duration of study treatment was a maximum of 48 weeks and follow-up visits were at 12, 24, and 48 weeks.

### Study Setting and Participants

Participants were recruited from 19 Sexual Health Clinics and/or HIV treatment centers throughout the United Kingdom between 19 March 2013 and 20 July 2015 after obtaining written informed consent. Detailed trial participant information and inclusion and exclusion criteria are given in the Supplementary data.

### Randomization and Masking

In stage I, patients were randomized in a 1:1:1:1 ratio using simple block randomization with random variable block length. Following the interim analysis, in stage II, eligible patients were randomized in an equal ratio to receive any of the promising doses or no intervention (control).

### Procedures

Telmisartan (20, 40, or 80 mg) was administered once daily with or without food. Patients randomized to telmisartan, 40 and 80 mg, attended 1 and 2 titration visits, respectively (see Supplementary data for data-collection methods). A summary of tests and investigations undertaken in each patient (see Supplementary Table 1) and the assessment methods used are provided in the Supplementary data.

### Outcomes

The primary outcome measure was a reduction in insulin resistance as measured by HOMA-IR, a measurable, validated surrogate marker of insulin resistance, in telmisartan treated arm(s) after 24 weeks of treatment in comparison with the control. Secondary outcome measures and their definitions are provided in the Supplementary data.

### Statistical Analysis

An original sample size of 336 evaluable patients was calculated based on a 1-sided type I error of 5% and a power of 90% on the assumption that the difference in HOMA-IR between baseline and 24 weeks of treatment (primary endpoint) was normally distributed with a common standard deviation and a standardized effect size of 0.545. Following interim analysis, the sample size was recalculated based on the number of arms taken forward into stage II and the rate of patient withdrawals observed.

### Primary and Secondary Outcome Analysis

Details of primary and secondary outcome analyses are given in the Supplementary data. Briefly, we evaluated 3 different doses of telmisartan against the control in stage I of the study and conducted an interim analysis that allowed ineffective doses to be eliminated quickly while a dose showing a reduction in HOMA-IR was taken forward. At the interim analysis, the sample standard deviation pooled across all 4 arms was determined and used to construct test statistics expressing the advantage of each of the 3 active treatments over control. The critical values for recommending that a treatment was taken to further testing at the interim and final analyses (−2.782 and −2.086, respectively) were chosen based on a method described by Magirr and Whitehead [32], generalizing the approach of Whitehead and Jaki [33].

For exploration of the secondary objectives, joint models [34, 35] were used to fully exploit the serial nature of these outcomes accounting for informative loss to follow-up and missingness. Details of secondary outcome analysis are given in the Supplementary data.

## RESULTS

**Figure 2.**
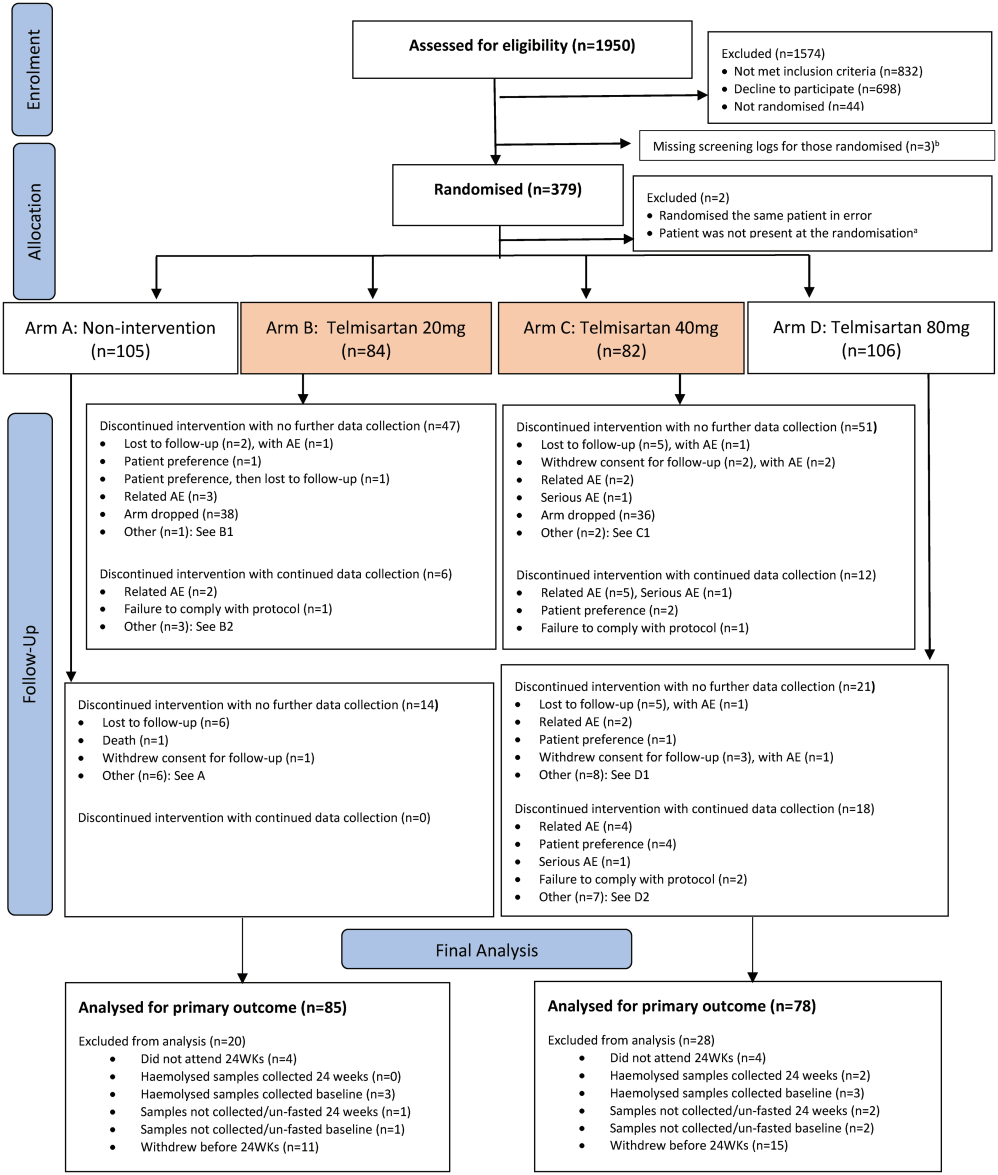
Trial profile. A: Patient moved to London—no forwarding address so unable to transfer patient; does not wish to continue the study due to organizational reasons; advice from study team following MRI incidental finding; to enter another clinical trial; eGFR less than 60 at screening; on nevirapine. B1: Concerned about leg swelling (not seen as an AE, saw GP, decided to stop drug). B2: Medication ran out 2 days before final visit; appointment missed—participant thought he should stop treatment before reschedule as canceled date 48 week; patient lost pills and failed to tell the research team until week 48 visit. C1: Patient has left the country; patient preference. C2: patients ran out of medication. D1: Patient developed a cough, wanted to discontinue study medication; Taking rampiril (GP’s orders); switch in ARV drug; not able to commit to study; eGFR low at baseline, did not meet eligibility criteria; did not return for appointment, did not respond to contacts; decided to become pregnant. D2: Patient ran out of medications and was due for his week 48 visit only a few days later; low eGFR; patient ran out of medication—new stock not collected; forgot to take them when on holiday abroad; ran out of medication; did not have enough telmisartan—could not come to pick up extra medication; patient usually takes telmisartan at nighttime—so did not take today before visit. ^a^One patient was randomized in arm B. The usual nurse is on extended leave and before leaving had asked for a number of patients to be randomized in her absence. Unfortunately, the cover nurse incorrectly assumed that she just randomize the patient immediately. The patient was not present at the time of randomization. ^b^Screening data are missing for 3 patients. Abbreviations: AE, adverse event; ARV, antiretroviral; eGFR, estimated glomerular filtration rate; GP, general practioner; MRI, magnetic resonance imaging.

A total of 377 patients were recruited (Figure 2) following the decision at interim analysis. Baseline characteristics were balanced across all 4 arms (Tables 1 and 2) including the cART regimens used. The study participants were predominantly males, with a comparable age, body mass index (BMI), CD4 cell count, baseline liver function, and full blood count in all arms (see Table 1 and Supplementary Tables 2–5). The median HOMA-IR was between 1.628 and 2.117 and comparable between all 4 arms (Table 2). There was also no difference in baseline values for the secondary outcome measures (Table 2).

**Table 1.** Baseline Characteristics of Patients by Treatment Group

	Treatment Arm			
Characteristic	Arm A (N = 105)^a^	Arm B (N = 84)	Arm C (N = 82)	Arm D (N = 106)^a^
Age, y				
Median (IQR)	47.2 (39.8–52.4)	46.0 (41.0–52.2)	47.9 (43.3–51.5)	45.8 (38.2–51.7)
[min–max]	[20.4–70.5]	[21.6–74.6]	[31.5–70.8]	[22.5–67.3]
Gender, n (%)				
Female	20 (19.0)	15 (17.9)	13 (15.9)	17 (16.0)
Male	85 (81.0)	69 (82.1)	69 (84.1)	89 (84.0)
BMI, n	103		81	
Median (IQR), kg/m^2^	25.4 (23.1–29.2)	25.6 (23.3–29.2)	26.3 (24.4–29.6)	25.4 (23.0–27.8)
[min–max], kg/m^2^	[16.7–42.0]	[18.8–52.2]	[16.7–46.3]	[17.9–43.7]
Systolic blood pressure, n			81	
Mean (SD), mm Hg	126.8 (13.9)	124.4 (14.2)	126.9 (14.3)	124.8 (15.4)
[min–max], mm Hg	[100.0–160.0]	[100.0–162.0]	[92.0–158.0]	[100.0–172.0]
Diastolic blood pressure, mmHg				
Mean (SD)	80.0 (10.7)	78.2 (11.2)	79.7 (9.9)	78.6 (11.1)
[min–max]	[60.0–122.0]	[56.0–107.0]	[54.0–102.0]	[55.0–107.0]
Respiratory rate, n	102	83	76	103
Mean (SD), breaths/min	15.6 (2.9)	15.9 (4.0)	16.0 (4.2)	16.5 (3.4)
[min–max], breaths/min	[10.0–28.0]	[10.0–41.0]	[10.0–37.0]	[10.0–28.0]
Waist circumference, n	101	83	79	102
Mean (SD), cm	93.5 (11.8)	94.6 (14.7)	97.1 (12.2)	93.0 (11.6)
[min–max], cm	[65.0–137.0]	[66.0–145.0]	[70.0–143.0]	[67.5–122.0]
Thigh circumference, n	101	82	77	102
Mean (SD), cm	50.8 (7.5)	52.3 (7.7)	51.8 (6.0)	49.6 (8.5)
[min–max], cm	[33.5–80.0]	[39.0–90.5]	[37.0–69.0]	[22.7–84.0]
eGFR,^b^ n	50	41	38	54
Mean (SD) [min–max] mL/min per 1.73m2	79.8 (13.6) [45.0–129.0]	79.9 (10.8) [60.0–105.0]	77.9 (10.5) [62.0 –107.6]	81.4 (14.5) [53.0 –‑ 122.0]
<60, n (%)	0 (0.0)	0 (0.0)	0 (0.0)	1 (0.9)
<90, n (%)	0 (1.0)	0 (0.0)	0 (0.0)	0 (0.0)
>60, n (%)	24 (22.9)	23 (27.4)	25 (30.5)	22 (20.8)
>90, n (%)	28 (26.7)	19 (22.6)	19 (23.2)	28 (26.4)

Abbreviations: BMI, body mass index; eGFR, estimated glomerular filtration rate; IQR, interquartile range; min–max, minimum–maximum; SD, standard deviation.

^a^Arm A and arm D were continued into the second stage of the trial; hence, the difference in sample size between these arms and the other 2 arms (arms B and C) of the trial.

^b^Some data are presented in both continuous and categorical form due to there being upper and lower limits of measurement.

**Table 2.** Baseline Values of Primary and Secondary Outcome Data by Treatment Group

	Treatment Arm			
Characteristic	Arm A (N = 105)	Arm B (N = 84)	Arm C (N = 82)	Arm D (N = 106)
Insulin, n	102	81	78	100
Median (IQR), pmol/L	54 (35–90)	57 (37–87)	61 (40–85)	51 (36.5–75.5)
[min–max], pmol/L	[11–279]	[21–319]	[21–432]	[21–454]
Glucose, n	104	83	80	104
Mean (SD), mmol/L	5.2 (0.5)	5.2 (0.58)	5.29 (0.7)	5.22 (0.54)
[min–max], mmol/L	[4.2–6.9]	[4.0–7.6]	[3.2–8.5]	[4.1–6.8]
NEFA, n	104	82	79	102
Median (IQR), mmol/L	0.42 (0.27–0.615)	0.385 (0.25–0.58)	0.35 (0.25–0.55)	0.40 (0.30–0.59)
[min–max], mmol/L	[0.08–1.21)	[0.07–1.08]	[0.05–0.84]	[0.08–1.21]
HOMA-IR, n	100	81	78	100
Median (IQR)	1.808 (1.120–2.903)	1.860 (1.208–3.508)	2.117 (1.223–3.297)	1.628 (1.175–.490)
[min–max]	[0.408–10.775]	[0.578–9.800]	[0.618–17.495]	[0.591–16.852]
QUICKI, n	100	81	78	100
Mean (SD)	0.117 (0.009)	0.116 (0.009)	0.116 (0.010)	0.118 (0.009)
[min–max]	[0.097–0.142]	[0.098–0.135]	[0.093–0.134]	[0.093–0.135]
Revised QUICKI, n	100	81	78	99
Mean (SD)	0.132 (0.017)	0.134 (0.019)	0.134 (0.019)	0.133 (0.016)
[min–max]	[0.101–0.184]	[0.100–0.211]	[0.100–0.212]	[0.096–0.178]
HDLc, n	104	82	79	103
Median (IQR), mmol/L	1.15 (0.9–1.4)	1.1 (1.0–1.4)	(0.9–1.4)	(1.0–1.4)
[min–max], mmol/L	[0.5–3.0]	[0.3–2.7]	[0.2–2.8]	[0.5–2.9]
Cholesterol, n	104	82	79	103
Mean (SD), mmol/L	5.01 (0.99)	5.0 (1.11)	4.83 (1.04)	4.97 (1.04)
[min–max], mmol/L	[2.2–8.2]	[2.6–8.3]	[2.5–7.1]	[2.9–7.64]
Triglycerides, n	104	82	79	103
Median (IQR), mmol/L	1.4 (1.0–2.0)	1.25 (0.9–1.8)	1.4 (1.0–1.9)	1.2 (0.9–1.8)
[min–max], mmol/L	[0.5–6.5]	[0.4–4.8]	[0.5–6.1]	[0.4–6.7]
LDLc, n	103	81	78	102
Mean (SD), mmol/L	3.1 (0.91)	3.14 (0.97)	2.97 (0.9)	3.12 (0.91)
[min–max], mmol/L	[1.1–6.4]	[1.1–6.2]	[0.8–5.2]	[0.8–5.6]
Adiponectin, n	104	82	78	101
Median (IQR), µg/mL	14.62 (10.11–20.50)	16.27 (12.05–21.84)	13.69 (9.22–20.31)	13.47 (8.28–18.51)
[min–max], µg/mL	[3.14–44.34]	[1.73–60.49]	[2.01–66.19]	[2.74–129.01]
Leptin, n	104	81	78	103
Median (IQR), pg/mL	4856.7 (1885.9–13 879)	4688.7 (2149–15 786)	5227.2 (2271.4–9710)	4492.2 (2126.5–10 192)
[min–max], pg/mL	[253.64–123 299]	[388.38–192 842]	[168.11–119 430]	[502.59–104 002]
IL-8, n	104	82	78	102
Median (IQR), pg/mL	17 (12.98–22.7)	14.38 (11.65–18.67)	16.32 (11.83–25.12)	18.57 (12.75–29.53)
[min–max], pg/mL	[5.67–744.98]	[5.18–166.7]	[6.04–187.86]	[4.51–368.69]
TNF-ɑ, n	103	82	78	101
Median (IQR), pg/mL	2.31 (1.69–3.49)	2.1 (1.74–2.49)	2.39 (1.71–2.99)	2.35 (1.77–3.09)
[min–max], pg/mL	[0.58–12.61]	[1.04–6.27]	[0.49–8.11]	[0.85–56.89]
Resistin, n	104	82	78	101
Median (IQR), pg/mL	5602.7 (3936.6–7998.5)	4790.2 (3713.7–6966.3)	5114 (3656.4–6861)	5684.9 (4590.9–8367.2)
[min–max], pg/mL	[1667.2–30 299]	[1288.2–20 357]	[1180–13 781]	[1607.7–19 692]
hs-CRP, n	104	82	78	103
Median (IQR), mg/L	2.24 (1.03–4.04)	1.4 (0.71–3.93)	1.32 (0.59–4.17)	1.32 (0.66–3.14)
[min–max], mg/L	[0.31–98.12]	[0.35–18.97]	[0.25–91.56]	[0.28–41.74]
NGAL, n	99	82	72	101
Median (IQR), pg/mL	5.99 (1.98–15.31)	5.59 (2.38–15.76)	6.30 (1.70–17.59)	5.48 (1.85–15.85)
[min–max], pg/mL	[0.46–160.79]	[0.59–325.54]	[0.49–282.04]	[0.61–160.50]
ACR, n	40	34	31	40
Median (IQR), mg/mmol	0.8 (0.4–3.6)	0.9 (0.6–1.8)	0.9 (0.5–2.0)	0.5 (0.4–1.65)
[min–max], mg/mmol	[0.2–37.0]	[0.2–8.5]	[0.2–7.4]	[0.3–38.8]

Abbreviations: ACR, albumin–creatinine ratio; HDLc, high-density-lipoprotein cholesterol; HOMA-IR, homeostasis model assessment of insulin resistance; hs-CRP, high-sensitivity C-reactive protein; IL-8, interleukin 8; IQR, interquartile range; LDLc, low-density-lipoprotein cholesterol; min–max, minimum–maximum; NEFA, nonesterified fatty acid; NGAL, neutrophil gelatinase-associated lipocalin; QUICKI, Quantitative Insulin Sensitivity Check Index; SD, standard deviation; TNF-ɑ, tumor necrosis factor ɑ.

**Figure 3.**
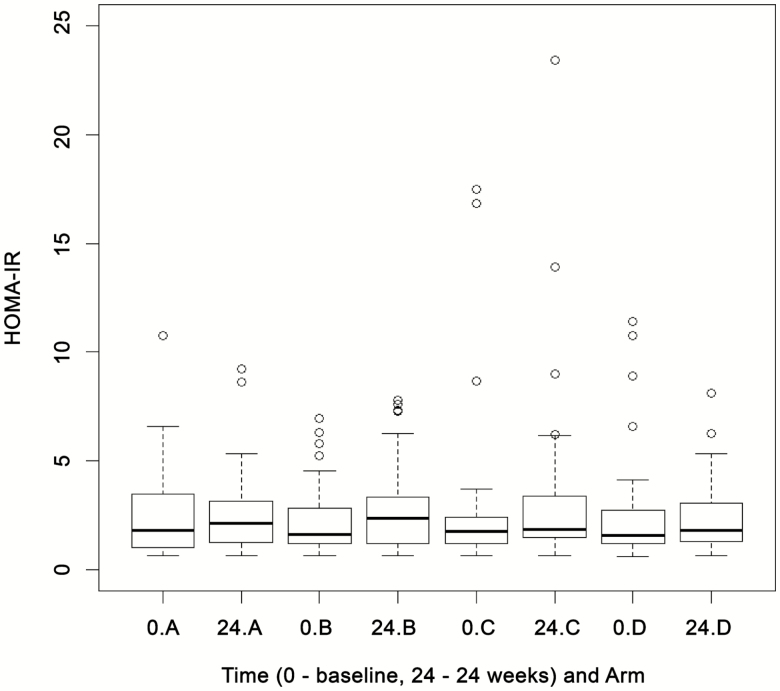
Box plots for HOMA-IR at baseline (0) and 24 weeks by treatment group at the interim analysis. “A” refers to the control arm; “B,” “C,” and “D” refer to arms treated with 20, 40, and 80 mg of telmisartan, respectively. Abbreviation: HOMA-IR, homeostasis model assessment of insulin resistance.

In stage I, 48, 49, 47, and 45 patients were randomized to arms A, B, C, and D, respectively (total n = 189); however, only the 154 patients who had a complete set of baseline and 24-week HOMA-IR data were included in the interim analysis. The t-statistic for arms B and C showed a positive value (ie, higher than 0), suggesting that there was no reduction in the HOMA-IR over control (arm A). These active-dose arms were thus dropped from stage II. As some improvement over control was detected for arm D (ie, the t-statistic was between 0 and −2.782), this arm was progressed to stage II of the trial and the patients were thereafter randomized between arm D (telmisartan, 80 mg) and arm A (control) (Figure 3) (details of summary statistics are given in the Supplementary data).

**Figure 4.**
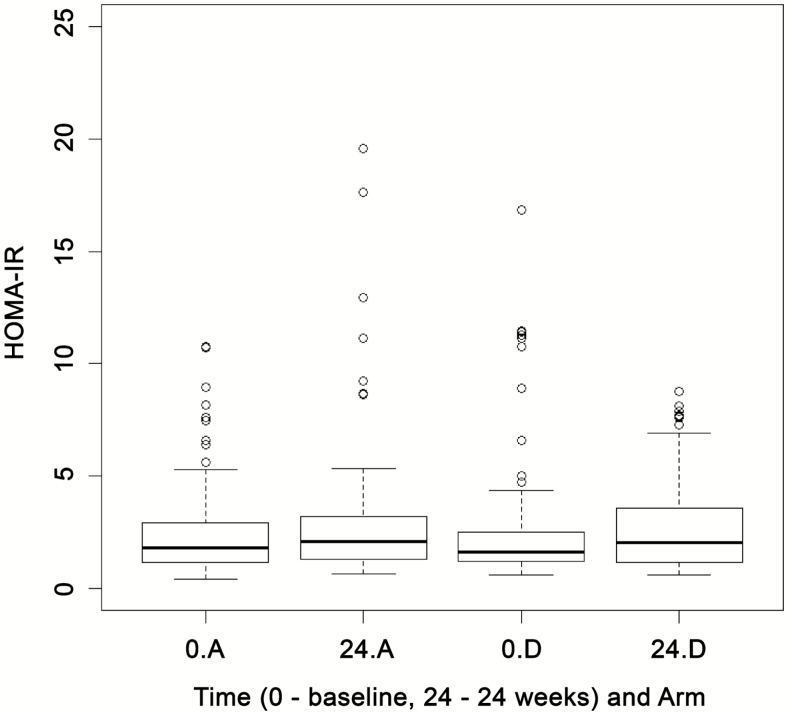
Box plots for HOMA-IR at baseline (0) and 24 weeks by treatment group at the final analysis for continued treatment groups. “A” refers to the control arm; “D” refers to the arm treated with 80 mg of telmisartan. Abbreviation: HOMA-IR, homeostasis model assessment of insulin resistance.

At the end of stage II, there were 105 and 106 patients randomized to arm A and arm D, respectively (total n = 211). The test statistic (−0.347) was not smaller than the critical value of −2.086; hence, it was concluded that there was no significant difference in HOMA-IR between arms A and D (treatment effect, 0.007; standard error [SE], 0.106) (Figure 4) (details of summary statistics are given in the Supplementary data). Post hoc analysis showed that the *P* value for the interaction between baseline HOMA-IR and arm D versus arm A treatment effect was .4714 (*P* > .05), indicating that a higher baseline HOMA-IR did not lead to a greater decline with telmisartan at 24 weeks (see Supplementary Table 8); this result remained the same even after adjustment for weight change, change in waist circumference, and statin use between baseline and 24 weeks (see Supplementary Tables 9–11). There were 26 (24.8%) and 21 (19.8%) individuals who showed a baseline HOMA-IR greater than 2.8 in arms A and D, respectively; again there was no significant difference in HOMA-IR (treatment effect of arm D compared with arm A, 0.277; 95% confidence interval [CI], −0.128 to 0.682; *P* = .17; see Supplementary Table 12) between the treatment and control arms at 24 weeks in this subset of individuals with a HOMA-IR greater than 2.8.

**Figure 5.**
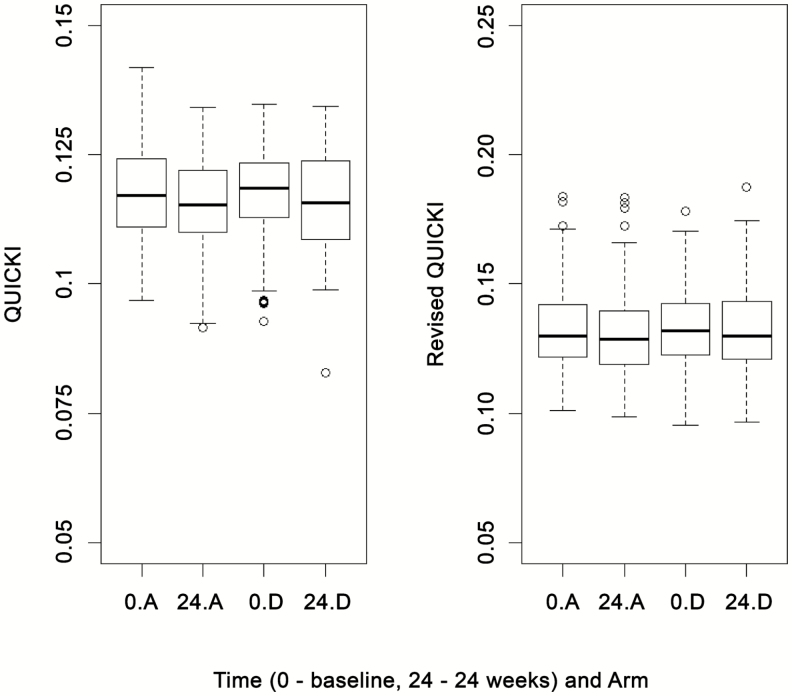
Box plots for QUICKI and revised QUICKI at baseline (0) and 24 weeks by treatment group. “A” refers to the control arm; “D” refers to arm treated with 80 mg of telmisartan. Abbreviation: QUICKI, Quantitative Insulin Sensitivity Check Index.

Two alternative measures of insulin sensitivity, the QUICKI and the revised QUICKI, were also used to investigate the effect of telmisartan. For both the QUICKI and revised QUICKI, the test statistics (0.4471 and 0.6882, respectively) were not smaller than the critical value (−2.086), suggesting no difference between arms A and D (treatment effects, 0.001 [SE, 0.001] and 0.002 [SE, 0.002], respectively) (Figure 5) (summary statistics are given in the Supplementary data).

The longitudinal profiles of secondary outcomes including HOMA-IR, QUICKI, and revised QUICKI at weeks 12, 24, and 48 for arm D compared with the control arm did not show any significant difference in HOMA-IR; however, the treatment effect of arm D compared with arm A for the longitudinal revised QUICKI was marginally significant (0.004; 95% CI, 0.000–0.008; *P* = .05), suggesting that telmisartan (80 mg) led to a small reduction in insulin resistance over a period of 48 weeks (see Supplementary data for all summary statistics).

There was no significant difference over 48 weeks between the treatment and control arms with any of the lipids or plasma biomarkers (see Supplementary data), apart from high-sensitivity C-reactive protein (hs-CRP), where significantly lower plasma levels were observed for patients on 80 mg telmisartan compared with controls (treatment effect, −0.222, 95% CI, −0.433 to −0.011; *P* = .04; see Supplementary data). A significant reduction in albumin–creatinine ratio (ACR) over 48 weeks was observed for the subgroup with an ACR greater than 3 mg/mmol (reduction in ACR, −0.665; 95% CI, −1.310 to −0.019; *P* = .04), suggesting a statistically significant but marginal treatment effect in arm D compared with the control arm. However, the estimated treatment effect on neutrophil gelatinase-associated lipocalin was not significant in any of the tertiles (see Supplementary data).

**Figure 6.**
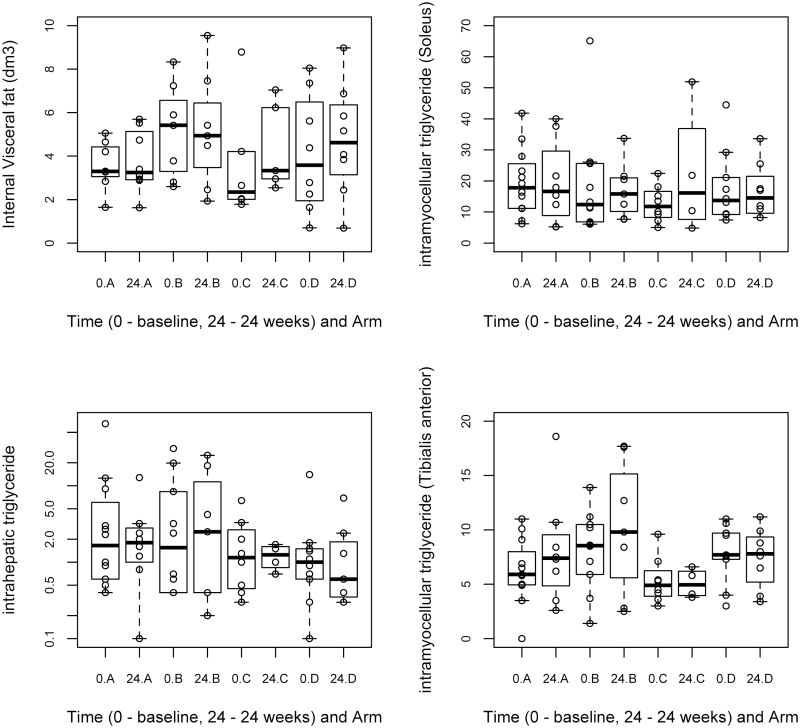
Box plots for the MRI and ^1^H MRS measurements at baseline (0) and 24 weeks by treatment group (with individual data points). “A” refers to the control arm; “B,” “C,” and “D” refer to arms treated with 20, 40, and 80 mg of telmisartan, respectively. Intrahepatic and intramyocellular triglyceride contents are expressed as ratios (CH2 relative to unsuppressed water and as CH2 relative to creatine signal, respectively). The vertical axis for intrahepatic triglyceride content has been plotted in logarithmic scale to include the extreme values. Abbreviations: MRI, magnetic resonance imaging; MRS, magnetic resonance spectroscopy.

A statistically significant difference in the intrahepatic triglyceride content was observed at 24 weeks between arm D and control (Figure 6) (1.714 mean reduction; 95% CI, −2.787 to −0.642; *P* = .005), although no difference was observed in internal visceral fat or limb fat (soleus and tibialis anterior) between the treatment and control groups (Figure 6) (see Supplementary data).

Diarrhea, fatigue, dizziness, and pruritus were the most common adverse reactions observed in more than 2% of patients who participated in the study. Twenty-one serious adverse events (SAEs) were reported from 19 (5.0%) patients, similarly distributed between all 4 arms (arm A, 4.8%; arm B, 3.6%; arm C, 4.9%; and arm D, 6.6%). There was no evidence of difference in SAEs between telmisartan-treated and control arms. We also did not observe any clinically important differences between those with drug-compliance data and those without (see Supplementary data).

## DISCUSSION

To the best of our knowledge, this is the first RCT to assess the effect of telmisartan on insulin resistance in HIV-infected individuals. We show that telmisartan (80 mg) did not reduce HOMA-IR, a surrogate marker of insulin resistance (primary outcome measure) over 24 weeks when compared with the control arm. Telmisartan also did not improve the insulin sensitivity indices QUICKI and revised QUICKI over 24 weeks. Longitudinal outcome analysis over 48 weeks also did not show any improvement in HOMA-IR and QUICKI, but there was a significant, but marginal, improvement in revised QUICKI (*P* = .05), serum hs-CRP, and urinary ACR (*P* = .04) in patients with microalbuminuria (ACR >3 mg/mmol). Telmisartan also significantly reduced liver fat over a period of 24 weeks but had no effect on total body or limb fat.

Only 3 studies evaluating telmisartan have been reported in patients with HIV. Two observational studies showed a reduction in insulin resistance with telmisartan [36, 37] in a limited number of patients (n = 18 and 13, respectively); the third was a single-arm open-label trial in 35 HIV-positive individuals [30], which failed to find a significant change in HOMA-IR at 24 weeks. In non–HIV-positive individuals, there have been a greater number of telmisartan studies. A meta-analysis comprising 2033 patients found telmisartan significantly reduced insulin resistance compared with other antihypertensives [23]. Some of these RCTs were conducted in patients with preexisting T2D or MS and were therefore naturally enriched with individuals who were highly insulin resistant at baseline [20, 21]. However, some trials have shown no beneficial effect of telmisartan even when the baseline HOMA-IR was high [24, 25]. Our trial had a median baseline HOMA-IR of 1.6 in the 80-mg telmisartan arm. A post hoc analysis of arm A versus arm D at 24 or 48 weeks showed that a higher baseline HOMA-IR did not lead to a greater decline with telmisartan. It should be noted that there is no universal threshold for defining insulin resistance and HOMA-IR varies between different ethnicities [38], but a threshold of HOMA-IR greater than 2.8 has been used to signify high insulin resistance. Approximately 25% of individuals in each arm of our trial had a HOMA-IR greater than 2.8; again, a stratified analysis in this group failed to show any reduction in HOMA-IR in the telmisartan arm in comparison to the control arm, but the sample size was small (arm A, n = 22; arm D, n = 14).

Although telmisartan did not result in a statistically significant reduction in HOMA-IR, it did result in a marginal beneficial effect on the revised QUICKI (*P* = .05), a surrogate index of insulin sensitivity that has recently been shown to be a better marker than HOMA-IR [39]. The revised QUICKI takes into account fasting serum nonesterified fatty acid levels, in addition to plasma glucose and serum insulin, and has better discriminatory power, particularly in nonobese individuals who present with mild insulin resistance [40]. Given that the TAILoR cohort had a median baseline BMI of 26.7 kg/m^2^, the revised QUICKI may have been a more sensitive indicator to measure the effect of telmisartan than HOMA-IR.

Telmisartan did not reduce visceral fat, consistent with a previous study in HIV-positive individuals [30], but did reduce liver fat in the 80-mg arm over a period of 24 weeks. However, given the small numbers, the findings have to be interpreted with caution. Nevertheless, intrahepatic fat has been suggested to be a better marker of metabolic disease than visceral fat [41] and may provide a better estimate of ectopic fatty acid deposition, which is one of the main reasons for the development of insulin resistance.

Telmisartan reduced hs-CRP, consistent with data from non-HIV clinical studies [21, 42] and meta-analysis [43]. This anti-inflammatory effect may be important given that hs-CRP is an independent predictor of CVD in HIV [44]. Our trial was not designed to investigate renal outcomes, but telmisartan (80 mg) reduced (*P* = .04) ACR over 48 weeks in patients with microalbuminuria (defined by ACR >3 mg/mmol) when compared with the control arm. This is in line with previous evidence of the renoprotective effect of telmisartan in patients with HIV [36].

There were no safety concerns with any of the doses of telmisartan, and the SAEs were similarly distributed between the treatment arms and the control arm. Our finding of no decrease in blood pressure even in normotensive patients was reassuring.

A major strength of the trial was the adaptive design utilised; this allowed testing of 3 different doses of telmisartan simultaneously and to drop 2 different “loser doses” so that a potential “winner dose” could be taken into the second stage of the study. It allowed us to take into account that the purpose of our study (reduction in insulin resistance) may have a different dose-response profile compared with hypertension, which is what telmisartan is licensed for. Additionally, this design also provided an opportunity to stop the study at the interim analysis stage if the required benefit was not identified with any of the doses. We used HOMA-IR as our primary outcome measure; ideally, we should have used the euglycemic-hyperglycemic clamp for ascertaining insulin resistance, but the invasiveness and complexity of undertaking this in a large-scale trial ruled it out. A 24-week time point was selected for the primary outcome and 48 weeks for the total duration of drug treatment based on data on non–HIV-positive patients. A longer duration of treatment may have been more ideal, but previous studies have shown that HOMA-IR changes within 4 weeks of starting ARV drugs [15].

In conclusion, this trial, which used a novel adaptive design, did not find a significant effect of telmisartan on reduction in HOMA-IR, the primary outcome measure, after 24 weeks of treatment. A longitudinal analysis over 48 weeks showed that telmisartan improved the revised QUICKI, hs-CRP, hepatic fat accumulation, and microalbuminuria. Although these changes were marginal, they are biologically plausible. Given the contradictory findings with telmisartan in populations with or without HIV, stratifying treatment with telmisartan using biomarkers may be a fruitful area for further research. To this end, the biological archive created as a result of the trial will serve as a valuable source for the identification and validation of novel biomarkers.

## Supplementary Data

Supplementary materials are available at *Clinical Infectious Diseases* online. Consisting of data provided by the authors to benefit the reader, the posted materials are not copyedited and are the sole responsibility of the authors, so questions or comments should be addressed to the corresponding author.

ciz589_suppl_Supplementary_Appendix

## Notes

***Author contributions.*** All authors meet the requirements for authorship. S. P., R. K.-D., M. G.-F., G. J. K., T. J., S. K., P. W., and M. P. designed the study. C. T. and C. S. set up the study sites. S. K. recruited participants. S. P., T. F., and G. J. K. conducted laboratory and MRI/MRS analyses, respectively. R. K.-D., T. J., M. G.-F., and P. W. analyzed the data. S. P., R. K.-D., M. G.-F., G. J. K., S. K., P. W., and M. P. interpreted the data. S. P., R. K.-D., C. T., C. S., M. G.-F., G. J. K., T. J., S. K., P. W., and M. P. wrote the report. All authors had access to the clinical study report, reviewed the article, and approved the submission of the article.

***Acknowledgments.*** The authors acknowledge the patients for their participation in the trial, the members of the trial steering committee and independent data and safety monitoring committee for their oversight, the clinical and nursing staff at the participating sites for recruitment, the North West Coast Clinical Research Network for providing support for clinical research delivery, and the University of Liverpool radiography staff for their help with magnetic resonance imaging/magnetic resonance spectroscopy (MRI/MRS) assessments. The authors also acknowledge the TAILoR Study Group Investigators: Jonathan Ainsworth (North Middlesex University Hospital National Health Service [NHS] Trust, London), David Chadwick (James Cook University Hospital, Middlesbrough), Mas Chaponda (St Helens Hospital, St Helens), Mayur Chauhan (Royal Victoria Infirmary, Newcastle upon Tyne), Duncan Churchill (Elton John Centre, Brighton and Sussex Hospitals NHS Trust, Brighton), Satyajit Das (Coventry and Warwickshire NHS Trust, Coventry), Mark Gompels (Southmead Hospital, Bristol), Elbushra Hereika (Royal Bournemouth and Christchurch Hospital, Bournemouth), Margaret Johnson (Royal Free London Hospital, London), Clifford Leen (Western General Hospital, Edinburgh), David Loay (George Elliot Hospital, Nuneaton), Fabiola Martin (Harrogate District Hospital, Harrogate), Jane Minton (Leeds Teaching Hospitals, St James University Hospital, Leeds), Barry Peters (Guys and St Thomas Hospital, London), Frank Post (Kings College Hospital, London), Gabriel Schembri (Manchester Centre for Sexual Health, Manchester), Jiten Vora (Royal Liverpool University Hospital, Liverpool), and John Whitehead (University of Lancaster, Lancaster).

***Disclaimer.*** The views expressed in this publication are those of the author(s) and not necessarily those of the Medical Research Council (MRC), NHS, National Institute for Health Research (NIHR), or the Department of Health.

***Financial support.*** This work was supported by the MRC NIHR Efficacy and Mechanism Evaluation (EME) Board (grant reference 10/60/37).

***Potential conflicts of interest.*** M. P. has received grants from the NIHR-MRC EME funding scheme, the MRC, and the European Union (EU) commission during the conduct of the study. He is program director for the MRC Clinical Pharmacology Training Scheme, which is funded jointly by the MRC and Industry (Roche, UCB Pharma, Novartis, and Eli Lilly). S. K. has received grants from the pharmaceutical industry (Merck, Gilead, ViiV Healthcare, Janssen) as support for the Liverpool HIV Drug Interactions website and for research projects. T. J. has received grants from the NIHR-Health Technology Assessment and funding from the pharmaceutical industry (Roche, Bayer, Novartis, Janssen, AstraZeneca, Baxalta) as a member of an EU project, as a member of an Independent Data Monitoring Committee, for a presentation at a conference, for a postdoctoral fellowship, and for a research project. P. W. has received income from Abbvie related to fees for the preparation of a Clinical Study Report for the SYCAMORE trial (EudraCT number, 2010-021141-41). All other authors report no potential conflicts. All authors have submitted the ICMJE Form for Disclosure of Potential Conflicts of Interest. Conflicts that the editors consider relevant to the content of the manuscript have been disclosed.
