# Supplementary material for: TAILoR (TelmisArtan and InsuLin Resistance in Human Immunodeficiency Virus [HIV]): An Adaptive-design, Dose-ranging Phase IIb Randomized Trial of Telmisartan for the Reduction of Insulin Resistance in HIV-positive Individuals on Combination Antiretroviral Therapy
Source: Clin Infect Dis. 2019 Jul 3;70(10):2062–72. doi: 10.1093/cid/ciz589 (PMC7201422; doi:10.1093/cid/ciz589)
Supplement: ciz589_suppl_Supplementary_Appendix [file ciz589_suppl_supplementary_appendix.docx]

**Additional file 1: Supplementary Appendix**

**TAILoR (TelmisArtan and InsuLin Resistance in HIV): An Adaptive Design Dose-Ranging Phase IIb Randomised Trial of Telmisartan for the Reduction of Insulin Resistance in HIV-Positive Individuals on Combination Antiretroviral Therapy**

Sudeep Pushpakom^1^, Ruwanthi Kolamunnage-Dona^2^, Claire Taylor^3^, Terry Foster^1^, Cath Spowart^3^, Marta Garcia-Finana^2^, Graham J Kemp^4^, Thomas Jaki^5^, Saye Khoo^1^, Paula Williamson^2^, Munir Pirmohamed^1†^ for the TAILoR Study Group

^1^Department of Molecular and Clinical Pharmacology, University of Liverpool, Liverpool, L69 3BX

^2^Department of Biostatistics, University of Liverpool, Liverpool, L69 3BX

^3^Clinical Trials Research Centre, University of Liverpool, Liverpool, L12 2AP

^4^Liverpool Magnetic Resonance Imaging Centre, University of Liverpool, Liverpool, L12 2AP

^5^Department of Mathematics and Statistics, Lancaster University, Lancaster, LA1 4YW

**†Corresponding Author:**

Professor Sir Munir Pirmohamed, Institute of Translational Medicine, University of Liverpool, Block A: Waterhouse Building, 1-5 Brownlow Street, Liverpool, L69 3GL

**Tel:** +44 151 794 5549

**Email:** [munirp@liverpool.ac.uk](mailto:munirp@liverpool.ac.uk)

**A summary of the protocol for the study has been published (the full protocol has also been uploaded)**

Pushpakom, S. P., Taylor, C., Kolamunnage-Dona, R., Spowart, C., Vora, J., Garcia-Finana, M., Kemp, G. J., Whitehead, J., Jaki, T., Khoo, S., Williamson, P., & Pirmohamed, M. (2015). Telmisartan and Insulin Resistance in HIV (TAILoR): protocol for a dose-ranging phase II randomised open-labelled trial of telmisartan as a strategy for the reduction of insulin resistance in HIV-positive individuals on combination antiretroviral therapy. *BMJ Open, 5*, e009566.

doi: 10.1136/bmjopen-2015-009566.

## **Trial Participants**

Participants were recruited from 19 Sexual Health Clinics and/or HIV treatment centres throughout the UK between March 2013 and July 2015. All participants gave written informed consent before taking part in the trial. All trial participants were adults with documented HIV infection, who had been receiving a stable cART for at least 6 months prior to randomisation.

### **Inclusion Criteria**

- Adult (age 18 or above) HIV-positive individuals receiving antiretroviral therapy containing

1. a boosted protease inhibitor (lopinavir/ritonavir, atazanavir/ritonavir, darunavir/ritonavir, fosamprenavir/ritonavir, saquinavir/ritonavir)
2. and/or efavirenz, rilpivirine, or etravirine for at least 6 months.

- Ability to give informed consent
- Willingness to comply with all study requirements

In relation to the antiretroviral therapy, the backbone was based on N(t)RTI, raltegravir or maraviroc. Patients on protease inhibitor monotherapy were also included if they met other criteria. Patients were excluded if they were on: (a) nevirapine or dolutegravir regimens, without concomitant boosted PIs; (b) elvitegravir which is usually administered in combination with cobicistat; and, (c) patients on unboosted atazanavir.

**Exclusion Criteria**

The exclusion criteria were as follows:

- Pre-existing diagnosis of type 1 or 2 diabetes (Fasting glucose > 7.2mmol/L or HbA1c ≥ 6.5% [48 mmol/mol] or abnormal OGTT or random plasma glucose ≥ 11mmol/l)
- Patients known to have consistently low blood pressure (pre-existing hypotension; A reading below a threshold of 100/60 mm Hg on three separate occasions)
- Patients with renal disease (eGFR<60 in the 6 months preceding randomisation)
- Patients with known untreated renal artery stenosis
- Patients with cholestasis, biliary obstructive disorders or severe hepatic impairment.
- Patients with evidence of an active, chronic hepatitis C infection (a previously cleared infection is not an exclusion)
- Patients who were on/ have been on hormone therapy (eg. growth hormone), anabolics (eg. testosterone) and insulin sensitisers (eg. Metformin) within 6 months preceding randomisation. Patients who were on hormonal contraception are eligible.
- Patients who were already on/ have been on other ARBs, ACE inhibitors, or direct renin inhibitors (e.g. aliskiren) within 4 weeks preceding randomisation.
- Those with suspected poor compliance
- Pregnant or lactating women
- Women of childbearing age unless using reliable contraception e.g. coil, barrier method, hormonal contraceptive that does not interact with their antiretroviral therapy
- Co-enrolment in other drug trials
- Patients who had participated in a trial of an IMP likely to influence insulin sensitivity, plasma insulin, glucose levels or plasma lipid levels within 6 months preceding randomisation.
- For the sub-cohort of patients undergoing MRI/MRS, normal MR exclusion criteria applied

**Randomisation and masking**

In stage I, patients were randomised in a 1:1:1:1 ratio using simple block randomisation with random variable block length. Following the interim analysis, in stage II, eligible patients were randomised in an equal ratio to receive any of the promising doses or no intervention (control). TAILoR was an open-labelled trial with the investigators and patients not blinded to the allocated treatment. However, allocation concealment was possible as participants were randomised using a secure web-based randomisation programme that was controlled centrally.

## **Data Collection**

The patients were asked to complete treatment diaries, detailing compliance, throughout the duration of treatment. A paper case report form (CRF) was used to collect patient data at each study visit. Paper CRFs were designed especially for the study in line with the trial protocol. The data was entered to a GCP compliant database (MACRO 3, Elsevier, Amsterdam, Netherlands) by trial staff at the CTRC. The configuration of the database was specific for the TAILoR trial, and it had built in validations on certain aspects of the trial data. A full audit trail was maintained. A summary of tests and investigations undertaken in each patient is provided in Supplementary Table 1.

**Procedures**

For each patient, three fasting blood samples and a urine sample were collected at 4 time points (baseline, 12, 24 and 48 weeks) during the trial. For the assessment of HOMA-IR (primary end point), serum insulin and plasma glucose were estimated by standard clinical chemistry methods. HOMA-IR was calculated using the equation: Fasting plasma insulin (mU/l) X fasting plasma glucose (mmol/l)/ 22.5. Other assessments included fasting serum nonesterified fatty acids (NEFA), Quantitative Insulin Sensitivity Check Index (QUICKI) and revised QUICKI [1], serum lipid profile, metabolic and inflammatory markers (adiponectin, leptin, resistin, IL-8, TNF-α, hs-CRP, urinary neutrophil gelatinase-associated lipocalin [NGAL]) and urine albumin creatinine ratio (ACR). In addition, visceral fat, intrahepatic fat and lower leg muscle fat (soleus and tibialis anterior) were measured at baseline and 24 weeks using magnetic resonance imaging (MRI) and proton magnetic resonance spectroscopy (^1^H MRS) [2].

Fasting serum nonesterified fatty acids (NEFA) were estimated using a colorimetric assay on a RX Daytona analyser (Randox Laboratories Limited, UK) and Quantitative Insulin Sensitivity Check Index (QUICKI) and revised QUICKI were estimated as previously reported [1]. Serum lipid profile and hs-CRP, and urine albumin creatinine ratio (ACR) were estimated using standard protocols. Metabolic and inflammatory biomarkers (adiponectin, leptin, resistin, IL-8 and TNF-α) and urinary neutrophil gelatinase-associated lipocalin (NGAL) were estimated using electrochemiluminescence-based immunoassays (Meso Scale Discovery, Rockville, Maryland, USA) according to the manufacturer’s protocols. Visceral fat, intrahepatic fat and lower leg muscle fat (soleus and tibialis anterior) were measured at baseline and after 24 weeks of telmisartan treatment using magnetic resonance imaging (MRI) and proton magnetic resonance spectroscopy (^1^H MRS) on a Siemens 1.5T Symphony scanner (Siemens, Erlangen Germany), using well-established methods [2].

## **Outcome measures**

### **Primary Outcome measure**

Reduction in insulin resistance (as measured by HOMA-IR) in telmisartan treated arm(s) after 24 weeks of treatment in comparison with control.

### **Secondary Outcome measures**

1. Reduction in insulin resistance (as measured by QUICKI and Revised QUICKI) in telmisartan treated arm(s) after 24 weeks of treatment in comparison with control.
2. Change in insulin resistance, measured longitudinally (HOMA-IR, QUICKI and revised QUICKI), in telmisartan treated arm(s) in comparison with the control arm.
3. Change in lipid profile at weeks 12, 24 and 48 (increase in HDL-c, reduction in total cholesterol, triglycerides and LDL-c) between telmisartan treated arm(s) and the control arm.
4. Change in plasma concentrations of biomarkers (adiponectin, leptin, IL-8, TNF-α, Resistin and hs-CRP) at weeks 12, 24 and 48 weeks between telmisartan treated arm(s) and the control arm.
5. Change in urinary biomarker levels (albumin:creatinine ratio, ACR; NGAL) at weeks 12, 24 and 48 weeks between telmisartan treated arm(s) and the control arm.
6. Change in body fat redistribution as measured by MRI/^1^H MRS at 24 weeks between telmisartan treated arm(s) and control arm (reduction in visceral fat, change in intrahepatic fat, change in lower leg muscle fat). This was conducted in small subset of patients recruited to each of the four arms.
7. Difference in expected and unexpected serious adverse events between different telmisartan treated dose arm(s) and the control arm.

#### **Primary outcome analysis**

In order to satisfy the primary objective, we evaluated three different doses of telmisartan against control in stage I of the study and conducted an interim analysis that allowed ineffective doses to be eliminated quickly while a dose showing a reduction in HOMA-IR was taken forward. At the interim analysis, the sample standard deviation pooled across all four arms was determined and used to construct test statistics expressing the advantage of each of the three active treatments over control. The critical values for recommending that a treatment was taken to further testing at the interim and final analyses (-2.782 and -2.086 respectively) were chosen based on a method described by Magirr et al [3], generalising the approach of Whitehead and Jaki [4]. These statistics were adjusted for the stratification factors (gender and ethnicity). The smallest of these test statistics was compared to the interim critical value (-2.782). Observing a test statistic below this value corresponded to a significant improvement in HOMA-IR score for the corresponding dose over control and would have led to this dose being immediately taken forward into phase 3, and to the trial being stopped. Any dose corresponding to a positive test statistic would have been dropped, and if all doses were dropped, the trial would also have been stopped. If some reduction in HOMA-IR over control was detected for at least one of the active doses (i.e. test statistic between 0 and -2.782), then the study continued after the interim analysis. At the final analysis, if the smallest comparative test statistic was below the final critical value (-2.086) then this dose would be recommended for further study. Adjustments were made to allow for any discrepancies between target and actual sample sizes while still preserving the one-sided type I error rate at 0.05.

#### **Secondary outcome analyses**

To explore the secondary objective of identifying longitudinal change in the expression of biomarkers in telmisartan treated arm(s) in comparison to controls, joint models [5, 6] were used to fully exploit the serial nature of these outcomes accounting for informative loss to follow up and missingness. The change in visceral fat, intrahepatic fat and limb fat at 24 weeks was compared across the three treatment groups and control using multiple linear regression. We used the same analysis as in the section Primary Outcome Analysis for the two alternative measures, QUICKI and revised QUICKI. Joint models included data from the dropped arms at the interim analysis and were fitted with longitudinal measurements from all four arms to adjust for informative dropout further and to account for changes of weight over time; bivariate joint models were fitted using joineRML package in R [7, 8]. Longitudinal measurements of urinary biomarkers were analysed using linear mixed effect models adjusting for age, weight change and gender which were included in the final model if found significant. Two additional ad hoc exploratory compliance adjusted analysis were undertaken for HOMA-IR at 24 weeks to address some selection bias. In addition, we undertook a post hoc analysis to determine whether the baseline HOMA-IR influenced the response to telmisartan; we also conducted a sub-group analysis in individuals with a baseline HOMA-IR>2.8 (an arbitrary threshold of HOMA-IR>2.8 to define high insulin resistance) to determine whether a response to telmisartan was observed in individuals with high insulin resistance.

Table S1: Schedule of trial procedures

| **Time** | **Pre T0** | **T0** | **T+2 week** | **T+4 weeks** | **T+12 weeks** | **T+24 weeks** | **T+48 weeks** |  |
| --- | --- | --- | --- | --- | --- | --- | --- | --- |
|  | **At each recruitment site** | **Randomisation/ Baseline*** | **Dose titration for 40/80mg arms (dose given 40mg)** | **Dose titration for 80mg arm (dose given 80mg)** | **Follow-up** | **Follow-up** | **End of treatment** | **Premature withdrawal of consent** |
| Database search to identify potential participants or clinic list review | Х |  |  |  |  |  |  |  |
| Information sheet provided to patient | X |  |  |  |  |  |  |  |
| Signed Informed consent |  | Х |  |  |  |  |  |  |
| Assessment of Eligibility Criteria by a medically qualified person |  | Х |  |  |  |  |  |  |
| Review of Medical History (including collection of most recent blood test results for Urea & electrolytes, eGFR, liver function, diabetes screening etc |  | Х** |  |  |  |  | X | Х |
| Review of Concomitant Medications |  | Х | Х | Х | Х | Х | Х | Х |
| Urine pregnancy test |  | Х |  |  | X | X |  |  |
| Randomisation |  | Х |  |  |  |  |  |  |
| Study Intervention |  | Х | Х | Х | Х | Х |  |  |
| Compliance with study intervention - patient diaries & pill counting |  |  | X | X | Х | Х | Х |  |
| Physical Exam - Complete |  | Х |  |  |  |  |  |  |
| Physical Exam - Symptom-Directed |  |  | Х | Х | Х | Х | X | Х |
| Height |  | Х |  |  |  |  |  |  |
| Weight |  | Х |  |  | Х | Х | Х | Х |
| Waist/thigh circumference |  | Х |  |  | Х | Х | Х | Х |
| Heart rate, blood pressure |  | Х | Х | Х | Х | Х | Х | Х |
| Collection of 3 fasting blood samples for bioanalysis |  | Х |  |  | Х | Х | Х | Х |
| Collection of urine sample |  | X |  |  | X | X | X | X |
| Assessment of Adverse Events |  |  | Х | Х | Х | Х | Х | Х |
| Consent for sub-study |  | X |  |  |  |  |  |  |
| MRI/MRS scan for sub-study |  | X |  |  |  | X |  |  |

**Baseline assessment and randomisation visit should be within 30 days of patient giving consent*

***Liver function and diabetes screening result only to be collected at baseline*

**Results**

**Baseline data**

Table S2: Baseline CD4 cell count & HIV viral load by treatment arm

| **Characteristic** | **Treatment Arm** | | | |
| --- | --- | --- | --- | --- |
|  | **Arm A**  **N=105** | **Arm B**  **N=84** | **Arm C**  **N=82** | **Arm D**  **N=106** |
| CD4 Cell count (cells/mm3), median (IQR) [min.–max.] | (n=102)  598 (480 - 756) [200 - 1400] | (n=83)  580 (425 - 792)  [131 - 1501] | (n=78)  582 (440 - 770)  [129 - 1467] | (n=101)  593 (427 – 783) [62 – 1674] |
| CD4 Cell count (%), mean (SD) [min.–max.] | (n=102)  32.1 (7.7)  [14.0 - 52.0] | (n=84)  29.2 (8.6)  [8.0 - 47.0] | (n=81)  30.1 (9.3)  [6.0 - 52.0] | (n=106)  30.5 (7.9)  [6.0 - 48.0] |
| HIV viral load <=50 copies/ml, n (%) | (n=102)  96 (91.4) | (n=82)  81 (96.4) | (n=82)  75 (91.5) | (n=104)  102 (96.2) |

Table S3: Baseline cART drug data by treatment arm

| **cART Regimen*** | **Treatment Arm** | | | | | | | |
| --- | --- | --- | --- | --- | --- | --- | --- | --- |
|  | **Arm A (n=105)** | | **Arm B (n=84)** | | **Arm C (n=82)** | | **Arm D (n=106)** | |
| **Boosted PI, n (%)** | 45 | (42.9) | 36 | (42.9) | 39 | (47.6) | 46 | (43.4) |
| ATV | 12 | (26.7) | 7 | (19.4) | 13 | (33.3) | 18 | (39.1) |
| DRV | 31 | (68.9) | 25 | (69.4) | 21 | (53.9) | 26 | (56.5) |
| Other^1^ | 2 | (4.4) | 4 | (11.1) | 5 | (12.8) | 2 | (4.4) |
| **NNRTI based, n (%)** | 55 | (52.4) | 42 | (50.0) | 38 | (46.3) | 54 | (50.9) |
| EFV | 46 | (83.6) | 38 | (90.5) | 30 | (79.0) | 42 | (77.8) |
| RPV | 4 | (7.3) | 3 | (7.1) | 6 | (15.8) | 7 | (13.0) |
| Other^2^ | 5 | (9.1) | 1 | (2.4) | 2 | (5.3) | 5 | (9.3) |
| **NRTI backbone^3^, n (%)** | 98 | (93.3) | 79 | (94.1) | 73 | (89.0) | 93 | (87.7) |
| TDF | 77 | (78.6) | 60 | (76.0) | 60 | (82.2) | 69 | (74.2) |
| ABC | 19 | (19.4) | 17 | (21.5) | 14 | (19.2) | 29 | (31.2) |
| 3TC | 20 | (20.4) | 19 | (24.1) | 13 | (17.8) | 25 | (26.9) |
| FTC | 77 | (78.6) | 58 | (73.4) | 57 | (78.1) | 61 | (65.6) |
| ZDV | 3 | (3.1) | 1 | (1.3) | 1 | (1.4) | 2 | (2.2) |
| **Integrase inhibitors, n (%)** | 4 | (3.8) | 8 | (9.5) | 3 | (3.7) | 4 | (3.8) |
| RAL | 4 | (100.0) | 6 | (75.0) | 3 | (100.0) | 4 | (100.0) |
| DTG | 0 | (0.0) | 1 | (12.5) | 0 | (0.0) | 0 | (0.0) |
| ELV | 0 | (0.0) | 1 | (12.5) | 0 | (0.0) | 0 | (0.0) |
| **Entry Inhibitors (MVC^4^), n (%)** | 2 | (1.9) | 1 | (1.2) | 2 | (2.4) | 0 | (0.0) |
| **PI monotherapy^5^, n (%)** | 4 | (3.8) | 5 | (6.0) | 6 | (7.3) | 11 | (10.4) |

**Integrase inhibitors, entry inhibitors and NRTIs were always co-prescribed with either boosted PIs and/or NNRTIs as specified in the inclusion criteria.*

*^1^Other boosted PIs prescribed were saquinavir, lopinavir and fosamprenavir*

*^2^Other NNRTIs used were nevirapine and etravirine*

*^3^The most common NRTI regimens used were TDF/FTC and ABC/3TC*

*^4^Only one entry inhibitor was used*

*^5^Darunavir was used as the PI in all but one patient*

*cART: Combination antiretroviral therapy; PI: Protease inhibitor; ATV: Atazanavir; DRV: Darunavir; NNRTI: Non-nucleoside reverse transcriptase inhibitor; EFV: Efavirenz; RPV: Rilpivirine; NRTI: Nucleoside reverse transcriptase inhibitor; TDF: Tenofovir disoproxil fumarate; ABC: Abacavir; 3TC: Lamivudine; FTC: Emtricitabine; ZDV: Zidovudine; RAL: Raltegravir; DTG: Dolutegravir; ELV: Elvitegravir; MVC: Maraviroc.*

Table S4: Baseline liver function by treatment arm

| **Characteristic** | **Treatment Arm** | | | |
| --- | --- | --- | --- | --- |
|  | **Arm A**  **N=105** | **Arm B**  **N=84** | **Arm C**  **N=82** | **Arm D**  **N=106** |
| ALT (iu/l), median (IQR) [min.–max.] | (n=92)  24.0 (17.5 - 33.5)  [9.0 - 80.0] | (n=75)  26.0 (19.0 - 36.0)  [6.0 - 142.0] | (n=77)  27.0 (20.0 - 35.0)  [10.0 - 84.0] | (n=96)  25.0 (18.0 - 38.0)  [8.0 - 305.0*] |
| ALP (iu/l), median (IQR) [min.–max.] | (n=103)  88.0 (71.0 - 107.0)  [30.0 - 371.0] | (n=83)  85.0 (72.0 - 113.0)  [40.0 - 403.0] | (n=74)  77.0 (62.0 – 98.0)  [32.0 - 179.0] | (n=102)  80.0 (65.0 - 100.0)  [35.0 - 309.0] |
| Albumin (g/l), mean (SD) [min.–max.] | (n=102)  43.7 (4.1)  [33.0 - 51.0] | (n=83)  44.3 (3.5)  [36.0 - 51.0] | (n=81)  44.2 (3.2)  [37.0 - 54.0] | (n=103)  44.7 (3.8)  [34.0 - 53.0] |
| Total protein (g/l), mean (SD) [min.–max.] | (n=72)  74.5 (5.3)  [63.0 - 87.0] | (n=59)  73.7 (4.0)  [66.0 - 84.0] | (n=59)  74.2 (4.6)  [65.0 - 85.0]` | (n=69)  73.4 (3.9)  [65.0 - 84.0] |
| Bilirubin (µmol/l), median (IQR) [min.–max.] ‡ | (n=100)  6.0 (5.0 - 9.5)  [2.0 - 67.0] | (n=78)  7.0 (5.0 - 9.0)  [3.0 - 82.0] | (n=76)  8.0 (6.0 - 12.0)  [3.0 - 100.0] | (n=98)  7.0 (5.0 - 14.0)  [2.0 - 68.0] |
|  |  |  |  |  |
| < 2, n (%) | 0 (0.0) | 1 (1.2) | 0 (0.0) | 0 (0.0) |
| < 3, n (%) | 3 (2.9) | 2 (2.4) | 2 (2.4) | 2 (1.9) |
| < 15, n (%) | 1 (1.0) | 2 (2.4) | 1 (1.2) | 4 (3.8) |

**confirmed correct*

*‡ Some data are presented in both continuous and categorical form due to there being upper and lower limits of measurement.*

Table S5: Baseline Full blood count by treatment arm

| **Characteristic** | **Treatment Arm** | | | |
| --- | --- | --- | --- | --- |
|  | **Arm A**  **N=105** | **Arm B**  **N=84** | **Arm C**  **N=82** | **Arm D**  **N=106** |
| Haematocrit (%), mean (SD) [min.–max.] | (n=70)  42.23 (3.29)  [34.2 - 53.0] | (n=49)  42.16 (6.51)  [5.1 - 51.0] | (n=50)  42.82 (3.18)  [34.6 – 49.9]` | (n=65)  42.46 (5.69)  [5.3 - 50.0] |
| Haemoglobin (g/dl), mean (SD) [min.–max.] | 143.77 (12.28)  [114 - 171] | (n=83)  144.16 (13.32)  [107 – 173] | 146.73 (12.15)  [114 - 177] | 145.72 (12.92)  [80 – 171] |
| Red blood cell count (10^12^/l), mean (SD) [min.–max.] | (n=98)  4.55 (0.44)  [3.47 - 5.63] | (n=79)  4.62 (0.44)  [3.23 - 5.59] | (n=77)  4.69 (0.41)  [3.68 - 6.06] | (n=97)  4.60 (0.44)  [3.10 - 5.79] |
| White blood cell count (10^9^/l), median (IQR) [min.–max.] | 5.98 (4.78 - 7.60)  [3.5 - 15.3] | 5.61 (4.8 - 7.2)  [3.0 - 13.8] | 5.56 (4.8 - 6.7)  [2.04 - 12.9] | (n=105)  5.7 (4.8 - 7.2)  [2.8 - 14.4] |
| Platelets (10^9^/l), median (IQR) [min.–max.] | (n=104)  231.5 (198.5 - 271.5)  [128 - 647] | 223.0 (193.0 - 252.0)  [46 - 406] | 216.5 (182.0 - 266.0)  [119 - 411] | 219.5 (185.0 - 262.0)  [106 - 368] |
| Mean Cell Volume (fl), mean (SD) [min.–max.] | (n=104)  93.40 (5.65)  [77.4 - 111.1] | 92.87 (5.48)  [73.6 - 104.0] | 92.47 (5.48)  [81.4 - 110.2] | 94.37 (5.33)  [81.0 - 111.0] |
| Mean Cell Haemoglobin (pg), mean (SD) [min.–max.] | (n=80)  31.49 (3.92)  [3.6 - 39.7] | (n=67)  31.35 (2.06)  [24.1 - 36.7] | (n=60)  31.34 (1.91)  [26.6 - 36.6] | (n=85)  31.35 (3.68)  [2.59 - 37.0] |
| Mean Cell Haemoglobin Concentration (g/dl), mean (SD) [min.–max.] | (n=68)  340.71 (11.83)  [309 – 366] | (n=53)  335.28 (12.97)  [302 – 366] | (n=53)  340.57 (13.03)  [315 – 367] | (n=65)  336.55 (12.48)  [293 – 357] |
| Neutrophils (10^9^/l), median (IQR) [min.–max.] | 3.15 (2.30 - 4.30)  [1.16 - 12.60] | 2.90 (2.24 - 3.90)  [1.10 - 9.70] | 2.90 (2.30 - 3.84)  [0.83 - 8.70] | 3.00 (2.10 - 3.80)  [0.88 - 10.00] |
| Lymphocytes (10^9^/l), median (IQR) [min.–max.] | 1.90 (1.60 - 2.55)  [0.89 - 4.10] | 2.13 (1.71 - 2.50)  [0.93 - 5.02] | 2.00 (1.62 - 2.31)  [0.53 - 3.70] | 1.91 (1.60 - 2.50)  [0.90 - 4.20] |
| Eosinophils (10^9^/l), median (IQR) [min.–max.] | (n=104)  0.11 (0.10 - 0.20)  [0.00 - 1.11] | (n=82)  0.10 (0.10 - 0.20)  [0.00 - 0.60] | 0.12 (0.10 - 0.20)  [0.00 - 0.41] | 0.12 (0.10 - 0.20)  [0.00 - 0.50] |
| Basophils (10^9^/l), median (IQR) [min.–max.] | (n=101)  0.02 (0.00 - 0.05)  [0.00 - 0.20] | (n=80)  0.00 (0.00 - 0.04)  [0.00 - 0.10] | (n=81)  0.00 (0.00 - 0.03)  [0.00 - 0.10] | (n=103)  0.01 (0.00 - 0.06)  [0.00 - 0.10] |
| Monocytes (10^9^/l), median (IQR) [min.–max.] | 0.50 (0.34 - 0.60)  [0.00 - 1.10] | 0.44 (0.30 - 0.60)  [0.19 - 1.20] | 0.48 (0.38 - 0.60)  [0.16 - 1.02] | 0.42 (0.31 - 0.60)  [0.10 - 1.49] |

**Summary statistics**

Table S6: Summary statistics for HOMA-IR at baseline and 24weeks by treatment group at the interim analysis

|  | **HOMA-IR at Baseline** | | | | **HOMA-IR at 24WKs** | | | |
| --- | --- | --- | --- | --- | --- | --- | --- | --- |
|  | **Arm A**  **(control)** | **Arm B**  **(20mg)** | **Arm C**  **(40mg)** | **Arm D**  **(80mg)** | **Arm A**  **(control)** | **Arm B**  **(20mg)** | **Arm C**  **(40mg)** | **Arm D**  **(80mg)** |
| N | 39 | 45 | 35 | 35 | 39 | 45 | 35 | 35 |
| Mean (SD)  [min. – max.] | 2.4 (2.0)  [0.6 - 10.8] | 2.3 (1.6)  [0.6 – 7.0] | 2.8 (3.9)  [0.6 - 17.5] | 2.6 (2.7)  [0.6 - 11.4] | 2.5 (1.9)  [0.6 - 9.2] | 2.7 (1.9)  [0.6 - 7.8] | 3.4 (4.4)  [0.6 - 23.5] | 2.5 (1.7)  [0.6 - 8.1] |
| Median (IQR) | 1.8 (1.0 – 3.5) | 1.6 (1.2 – 2.8) | 1.8 (1.2 – 2.5) | 1.6 (1.2 – 2.9) | 2.1 (1.2 – 3.2) | 2.4 (1.2 – 3.3) | 1.8 (1.4 – 3.4) | 1.8 (1.3 – 3.1) |
| N randomised | 48 | 49 | 47 | 45 | 48 | 49 | 47 | 45 |

Table S7: Summary statistics for HOMA-IR at baseline and 24 weeks by treatment group at the final analysis

|  | **HOMA-IR at Baseline** | | **HOMA-IR at 24weeks** | |
| --- | --- | --- | --- | --- |
|  | **Arm A**  **(control)** | **Arm D**  **(80mg)** | **Arm A**  **(control)** | **Arm D**  **(80mg)** |
| N | 100 (95.2%) | 99 (93.4%) | 89 (84.8%) | 81 (76.4%) |
| Mean (SD)  [min. – max.] | 2.49 (2.08)  [0.41 - 10.78] | 2.54 (2.81)  [0.59 - 16.85] | 2.99 (3.25)  [0.62 - 19.6] | 2.72 (2.15)  [0.60 - 8.77] |
| Median (IQR) | 1.81 (1.12 - 2.90) | 1.62 (1.18 - 2.48) | 2.09 (1.29 - 3.17) | 1.99 (1.15 - 3.23) |
| Missing | 5 (4.8%) | 7 (6.6%) | 16 (15.2%) | 25 (23.6%) |
| N randomised | 105 | 106 | 105 | 106 |

**Table S8: Model estimates for HOMA-IR at 24 weeks including the interaction between baseline HOMA-IR and treatment Arm D vs A**

| **Variable** | **Parameter Estimate** | **Standard Error** | **95% Confidence Limits** | | **P-value** |
| --- | --- | --- | --- | --- | --- |
| Intercept | 0.460 | 0.142 | 0.179 | 0.740 | 0.0015 |
| Log HOMA-IR at baseline | 0.541 | 0.108 | 0.327 | 0.754 | <0.0001 |
| Ethnicity (Non-Black) | 0.011 | 0.132 | -0.249 | 0.272 | 0.9324 |
| Arm D versus Arm A | -0.059 | 0.140 | -0.335 | 0.217 | 0.6747 |
| Baseline Log HOMA-IR* Arm D versus Arm A | 0.115 | 0.1594 | -0.199 | 0.429 | 0.4714 |

**Table S9: Model estimates for HOMA-IR at 24 weeks including the interaction between baseline HOMA-IR and treatment Arm D vs A, adjusted for weight changes**

| **Variable** | **Parameter Estimate** | **Standard Error** | **95% Confidence Limits** | | **P-value** |
| --- | --- | --- | --- | --- | --- |
| Intercept | 0.410 | 0.140 | 0.133 | 0.687 | 0.0040 |
| Log HOMA-IR at baseline | 0.523 | 0.106 | 0.314 | 0.732 | <0.0001 |
| Ethnicity (Non-Black) | 0.059 | 0.131 | -0.199 | 0.317 | 0.6536 |
| Weight change | 0.055 | 0.017 | 0.020 | 0.089 | 0.0020 |
| Arm D versus Arm A | -0.094 | 0.137 | -0.365 | 0.176 | 0.4906 |
| Baseline Log HOMA-IR* Arm D versus Arm A | 0.167 | 0.156 | -0.141 | 0.475 | 0.2860 |

**Table S10: Model estimates for HOMA-IR at 24 weeks adjusted for waist circumference**

| **Variable** | **Parameter Estimate** | **Standard Error** | **95% Confidence Limits** | | **Pr > \|t\|** |
| --- | --- | --- | --- | --- | --- |
| Intercept | 0.464 | 0.141 | 0.186 | 0.0012 | 0.741 |
| Log HOMA-IR at baseline | 0.578 | 0.082 | 0.416 | 0<0.0001 | 0.740 |
| Ethnicity (Non-Black) | 0.007 | 0.135 | -0.261 | 0.9613 | 0.274 |
| Change in waist circumference | 0.002 | 0.009 | -0.015 | 0.8056 | 0.019 |
| Arm D versus Arm A | -0.016 | 0.109 | -0.231 | 0.8869 | 0.200 |

**Table S11: Model estimates for HOMA-IR at 24 weeks adjusted for statin use**

| **Variable** | **Parameter Estimate** | **Standard Error** | **95% Confidence Limits** | | **Pr > \|t\|** |
| --- | --- | --- | --- | --- | --- |
| Intercept | 0.459 | 0.141 | 0.180 | 0.0014 | 0.738 |
| Log HOMA-IR at baseline | 0.575 | 0.083 | 0.411 | 0<0.0001 | 0.738 |
| Ethnicity (Non-Black) | 0.002 | 0.136 | -0.267 | 0.9882 | 0.271 |
| Waist change | 0.002 | 0.009 | -0.015 | 0.8021 | 0.019 |
| Statin use | 0.066 | 0.153 | -0.236 | 0.6675 | 0.368 |
| Arm D versus Arm A | -0.014 | 0.109 | -0.230 | 0.8956 | 0.201 |

Table S12: Model estimates for HOMA-IR at 24 weeks for the subset of individuals with HOMA-IR>2.8, adjusted for waist circumference

| **Variable** | **Parameter Estimate** | **Standard Error** | **95% Confidence Limits** | | **Pr > \|t\|** |
| --- | --- | --- | --- | --- | --- |
| Intercept | 0.072 | 0.428 | -0.800 | 0.8671 | 0.944 |
| Log HOMA-IR at baseline | 0.492 | 0.221 | 0.041 | 0.0335 | 0.944 |
| Ethnicity (Non-Black) | 0.419 | 0.246 | -0.082 | 0.0983 | 0.921 |
| Waist change | 0.032 | 0.016 | -0.001 | 0.0543 | 0.064 |
| Arm D versus Arm A | 0.277 | 0.198 | -0.128 | 0.1725 | 0.682 |

Table S13: Summary statistics for QUICKI at baseline and 24 weeks by treatment group

|  | **QUICKI at Baseline** | | **QUICKI at 24weeks** | |
| --- | --- | --- | --- | --- |
|  | **Arm A**  **(control)** | **Arm D**  **(80mg)** | **Arm A**  **(control)** | **Arm D**  **(80mg)** |
| N | 100 (95.2%) | 99 (93.4%) | 89 (84.8%) | 81 (76.4%) |
| Mean (SD)  [min.–max.] | 0.117 (0.0092)  [0.097 - 0.142] | 0.118 (0.0092)  [0.093 - 0.135] | 0.115 (0.0093)  [0.092 - 0.134] | 0.116 (0.0099)  [0.099 - 0.134] |
| Median (IQR) | 0.117 (0.111 -0.124) | 0.119 (0.113 - 0.123) | 0.115 (0.110 - 0.122) | 0.116 (0.110 - 0.124) |
| Missing | 5 (4.8%) | 7 (6.6%) | 16 (15.2%) | 25 (23.6%) |
| N randomised | 105 | 106 | 105 | 106 |

Table S14: Summary statistics for Revised-QUICKI at baseline and 24 weeks by treatment group

|  | **Revised-QUICKI at Baseline** | | **Revised-QUICKI at 24weeks** | |
| --- | --- | --- | --- | --- |
|  | **Arm A**  **(control)** | **Arm D**  **(80mg)** | **Arm A**  **(control)** | **Arm D**  **(80mg)** |
| N | 100 (95.2%) | 98 (92.4%) | 88 (83.8%) | 81 (76.4%) |
| Mean (SD)  [min.–max.] | 0.132 (0.0168)  [0.101 - 0.184] | 0.133 (0.0156)  [0.096 - 0.178] | 0.132 (0.0176)  [0.099 - 0.183] | 0.134 (0.0174)  [0.103 - 0.187] |
| Median (IQR) | 0.13 (0.122 - 0.142) | 0.132 (0.123 - 0.143) | 0.129 (0.119 - 0.140) | 0.131 (0.121 - 0.143) |
| Missing | 5 (4.8%) | 8 (7.5%) | 17 (16.2%) | 25 (23.6%) |
| N randomised | 105 | 106 | 105 | 106 |

## **Analysis of secondary outcome measures**

**Longitudinal outcomes of HOMA-IR, QUICKI and Revised QUICKI**

Table S15: Model* estimates for longitudinal measurements of HOMA-IR adjusted for weight changes and informative dropout

| **Component** | **Parameter** | **Estimate** | **95% CI Lower limit** | **95% CI Upper limit** | **P-value** |
| --- | --- | --- | --- | --- | --- |
| Longitudinal Marker | Intercept | 0.420 | 0.234 | 0.606 | <0.0001 |
|  | Time | 0.003 | 0.001 | 0.006 | 0.0159 |
|  | Baseline marker | 0.669 | 0.589 | 0.748 | <0.0001 |
|  | Treatment D vs A | -0.083 | -0.247 | 0.082 | 0.3243 |
|  | Ethnicity | -0.107 | -0.247 | 0.033 | 0.1343 |
| Longitudinal  Weight | Intercept | 1.047 | -1.131 | 3.226 | 0.3459 |
|  | Time | 0.022 | -0.002 | 0.045 | 0.0686 |
|  | Baseline weight | 0.987 | 0.965 | 1.008 | <0.0001 |
|  | Treatment D vs A | 0.117 | -0.842 | 1.076 | 0.8111 |
|  | Ethnicity | -0.246 | -1.055 | 0.562 | 0.5504 |
| Dropout | Treatment D vs A | 0.081 | -0.716 | 0.877 | 0.8427 |
|  | Ethnicity | -0.036 | -0.847 | 0.775 | 0.9300 |
| Association parameters | Marker | -0.262 | -1.433 | 0.909 | 0.6611 |
|  | Weight | 0.044 | -0.208 | 0.296 | 0.7337 |

**The bivariate joint model also included data from two dropped arms (B and C) to adjust for informative dropout further and assumed that these patients completed the trial as planned.*

**Table S16: Model* estimates for longitudinal measurements of HOMA-IR including the interaction between baseline HOMA-IR and Arm D vs A adjusted for weight changes and informative dropout**

| **Component** | **Parameter** | **Estimate** | **95%Lower** | **95%Upper** | **P-value** |
| --- | --- | --- | --- | --- | --- |
| Longitudinal  Log (HOMA-IR) | Intercept | 0.433 | 0.236 | 0.629 | <0.0001 |
|  | Time | 0.003 | 0.001 | 0.006 | 0.0208 |
|  | Log HOMA-IR at baseline | 0.661 | 0.555 | 0.766 | <0.0001 |
|  | Treatment B vs A | -0.109 | -0.361 | 0.142 | 0.3947 |
|  | Treatment C vs A | -0.035 | -0.283 | 0.214 | 0.7847 |
|  | Treatment D vs A | -0.107 | -0.338 | 0.123 | 0.3610 |
|  | Ethnicity | -0.099 | -0.247 | 0.049 | 0.1887 |
|  | Baseline Log HOMA-IR*Arm B versus Arm A | 0.015 | -0.218 | 0.248 | 0.8999 |
|  | Baseline Log HOMA-IR*Arm C versus Arm A | 0.043 | -0.146 | 0.231 | 0.6559 |
|  | Baseline Log HOMA-IR*Arm D versus Arm A | 0.035 | -0.189 | 0.259 | 0.7569 |
| Longitudinal  Weight | Intercept | 1.020 | -1.155 | 3.195 | 0.3580 |
|  | Time | 0.021 | -0.002 | 0.045 | 0.0746 |
|  | Baseline weight | 0.987 | 0.966 | 1.008 | <0.0001 |
|  | Treatment B vs A | 0.461 | -0.545 | 1.467 | 0.3694 |
|  | Treatment C vs A | 0.692 | -0.362 | 1.746 | 0.1981 |
|  | Treatment D vs A | 0.115 | -0.841 | 1.071 | 0.8135 |
|  | Ethnicity | -0.249 | -1.069 | 0.570 | 0.5510 |
| Dropout | Treatment B vs A | -0.353 | -1.269 | 0.562 | 0.4497 |
|  | Treatment C vs A | -0.074 | -0.991 | 0.842 | 0.8734 |
|  | Treatment D vs A | 0.082 | -0.717 | 0.882 | 0.8405 |
|  | Ethnicity | -0.036 | -0.850 | 0.778 | 0.9307 |
| Association parameters | Marker | -0.290 | -1.506 | 0.926 | 0.6403 |
|  | Weight | 0.046 | -0.208 | 0.300 | 0.7228 |

**The bivariate joint model is fitted including the interaction term* $HOMAIR\_0 * treatment$*. This model included longitudinal HOMA-IR measurements from all 4 arms to adjust for informative dropout further and accounting for changes of weight over time.*

Table S17: Model* estimates for longitudinal measurements of QUICKI adjusted for weight changes and informative dropout

| **Component** | **Parameter** | **Estimate** | **95% CI Lower limit** | **95% CI Upper limit** | **P-value** |
| --- | --- | --- | --- | --- | --- |
| Longitudinal Marker | Intercept | 0.039 | 0.029 | 0.048 | <0.0001 |
|  | Time | 0.000 | 0.000 | 0.000 | 0.0126 |
|  | Baseline marker | 0.649 | 0.572 | 0.726 | <0.0001 |
|  | Treatment D vs A | 0.001 | -0.001 | 0.003 | 0.3426 |
|  | Ethnicity | 0.001 | -0.001 | 0.003 | 0.2102 |
| Longitudinal  Weight | Intercept | 1.091 | -1.066 | 3.248 | 0.3217 |
|  | Time | 0.021 | -0.002 | 0.045 | 0.0755 |
|  | Baseline weight | 0.986 | 0.965 | 1.007 | <0.0001 |
|  | Treatment D vs A | 0.135 | -0.823 | 1.093 | 0.7823 |
|  | Ethnicity | -0.253 | -1.053 | 0.546 | 0.5344 |
| Dropout | Treatment D vs A | 0.082 | -0.716 | 0.879 | 0.8408 |
|  | Ethnicity | -0.037 | -0.835 | 0.762 | 0.9279 |
| Association parameters | Marker | 18.535 | -75.599 | 112.668 | 0.6996 |
|  | Weight | 0.042 | -0.210 | 0.295 | 0.7428 |

**The bivariate joint model also included data from two dropped arms (B and C) to adjust for informative dropout further and assumed that these patients completed the trial as planned.*

Table S18: Model* estimates for longitudinal measurements of Revised QUICKI adjusted for weight changes and informative dropout

| **Component** | **Parameter** | **Estimate** | **95% CI Lower limit** | **95% CI Upper limit** | **P-value** |
| --- | --- | --- | --- | --- | --- |
| Longitudinal Marker | Intercept | 0.058 | 0.046 | 0.070 | <0.0001 |
|  | Time | 0.000 | 0.000 | 0.000 | 0.0402 |
|  | Baseline marker | 0.550 | 0.473 | 0.627 | <0.0001 |
|  | Treatment D vs A | 0.004 | 0.000 | 0.008 | 0.0510 |
|  | Ethnicity | 0.001 | -0.002 | 0.004 | 0.5071 |
| Longitudinal  Weight | Intercept | 1.039 | -1.087 | 3.164 | 0.3382 |
|  | Time | 0.020 | -0.003 | 0.044 | 0.0899 |
|  | Baseline weight | 0.987 | 0.966 | 1.009 | <0.0001 |
|  | Treatment D vs A | 0.187 | -0.748 | 1.123 | 0.6945 |
|  | Ethnicity | -0.250 | -1.039 | 0.539 | 0.5340 |
| Dropout | Treatment D vs A | 0.078 | -0.726 | 0.881 | 0.8496 |
|  | Ethnicity | -0.087 | -0.845 | 0.671 | 0.8218 |
| Association parameters | Marker | 10.682 | -47.031 | 68.395 | 0.7168 |
|  | Weight | 0.028 | -0.206 | 0.262 | 0.8153 |

**The bivariate joint model also included data from two dropped arms (B and C) to adjust for informative dropout further and assumed that these patients completed the trial as planned.*

Longitudinal outcomes of lipids

Table S19: Model* estimates for longitudinal measurements of HDL-c adjusted for weight changes and informative dropout

| **Component** | **Parameter** | **Estimate** | **95% CI Lower limit** | **95% CI Upper limit** | **P-value** |
| --- | --- | --- | --- | --- | --- |
| Longitudinal Marker | Intercept | 0.021 | -0.034 | 0.076 | 0.4583 |
|  | Time | 0.000 | -0.001 | 0.001 | 0.6088 |
|  | Baseline marker | 0.878 | 0.833 | 0.924 | <0.0001 |
|  | Treatment D vs A | 0.001 | -0.046 | 0.047 | 0.9816 |
|  | Ethnicity | -0.018 | -0.063 | 0.028 | 0.4455 |
| Longitudinal  Weight | Intercept | 0.285 | -2.072 | 2.641 | 0.8129 |
|  | Time | 0.019 | -0.007 | 0.045 | 0.1537 |
|  | Baseline weight | 0.994 | 0.971 | 1.017 | <0.0001 |
|  | Treatment D vs A | 0.075 | -0.975 | 1.125 | 0.8881 |
|  | Ethnicity | -0.067 | -0.945 | 0.811 | 0.8817 |
| Dropout | Treatment D vs A | 0.329 | -0.545 | 1.204 | 0.4608 |
|  | Ethnicity | -0.216 | -1.078 | 0.646 | 0.6232 |
| Association parameters | Marker | -0.638 | -5.534 | 4.259 | 0.7986 |
|  | Weight | -0.046 | -0.244 | 0.151 | 0.6465 |

**The bivariate joint model also included data from two dropped arms (B and C) to adjust for informative dropout further and assumed that these patients completed the trial as planned.*

Table S20: Model* estimates for longitudinal measurements of cholesterol adjusted for weight changes and informative dropout

| **Component** | **Parameter** | **Estimate** | **95% CI Lower limit** | **95% CI Upper limit** | **P-value** |
| --- | --- | --- | --- | --- | --- |
| Longitudinal Marker | Intercept | 1.325 | 0.965 | 1.684 | <0.0001 |
|  | Time | 0.000 | -0.002 | 0.003 | 0.8473 |
|  | Baseline marker | 0.738 | 0.683 | 0.794 | <0.0001 |
|  | Treatment D vs A | 0.013 | -0.173 | 0.199 | 0.8904 |
|  | Ethnicity | -0.031 | -0.201 | 0.139 | 0.7219 |
| Longitudinal  Weight | Intercept | 0.218 | -2.106 | 2.541 | 0.8544 |
|  | Time | 0.021 | -0.005 | 0.046 | 0.1115 |
|  | Baseline weight | 0.995 | 0.972 | 1.017 | <0.0001 |
|  | Treatment D vs A | 0.076 | -0.954 | 1.105 | 0.8854 |
|  | Ethnicity | -0.090 | -0.982 | 0.802 | 0.8433 |
| Dropout | Treatment D vs A | 0.324 | -0.549 | 1.197 | 0.4666 |
|  | Ethnicity | -0.215 | -1.028 | 0.598 | 0.6042 |
| Association parameters | Marker | -0.040 | -1.197 | 1.118 | 0.9466 |
|  | Weight | -0.043 | -0.249 | 0.163 | 0.6807 |

**The bivariate joint model also included data from two dropped arms (B and C) to adjust for informative dropout further and assumed that these patients completed the trial as planned.*

Table S21: Model* estimates for longitudinal measurements of triglycerides adjusted for weight changes and informative dropout

| **Component** | **Parameter** | **Estimate** | **95% CI Lower limit** | **95% CI Upper limit** | **P-value** |
| --- | --- | --- | --- | --- | --- |
| Longitudinal Marker | Intercept | 0.018 | -0.091 | 0.128 | 0.7450 |
|  | Time | 0.001 | -0.001 | 0.002 | 0.4220 |
|  | Baseline marker | 0.779 | 0.713 | 0.845 | <0.0001 |
|  | Treatment D vs A | 0.030 | -0.056 | 0.116 | 0.4885 |
|  | Ethnicity | 0.005 | -0.089 | 0.099 | 0.9156 |
| Longitudinal  Weight | Intercept | 0.646 | -1.688 | 2.980 | 0.5876 |
|  | Time | 0.021 | -0.004 | 0.046 | 0.0962 |
|  | Baseline weight | 0.990 | 0.967 | 1.012 | <0.0001 |
|  | Treatment D vs A | 0.043 | -0.953 | 1.039 | 0.9332 |
|  | Ethnicity | -0.088 | -0.948 | 0.772 | 0.8412 |
| Dropout | Treatment D vs A | 0.324 | -0.547 | 1.195 | 0.4656 |
|  | Ethnicity | -0.219 | -1.031 | 0.593 | 0.5967 |
| Association parameters | Marker | -0.392 | -3.779 | 2.996 | 0.8207 |
|  | Weight | -0.035 | -0.269 | 0.199 | 0.7722 |

**The bivariate joint model also included data from two dropped arms (B and C) to adjust for informative dropout further and assumed that these patients completed the trial as planned.*

**Table S22: Model* estimates for longitudinal measurements of LDL-c adjusted for weight changes and informative dropout**

| **Component** | **Parameter** | **Estimate** | **95% CI Lower limit** | **95% CI Upper limit** | **P-value** |
| --- | --- | --- | --- | --- | --- |
| Longitudinal Marker | Intercept | 0.787 | 0.539 | 1.035 | <0.0001 |
|  | Time | 0.000 | -0.002 | 0.002 | 0.9532 |
|  | Baseline marker | 0.745 | 0.688 | 0.802 | <0.0001 |
|  | Treatment D vs A | 0.000 | -0.145 | 0.144 | 0.9946 |
|  | Ethnicity | 0.029 | -0.108 | 0.165 | 0.6820 |
| Longitudinal  Weight | Intercept | 0.223 | -2.143 | 2.589 | 0.8536 |
|  | Time | 0.021 | -0.005 | 0.048 | 0.1170 |
|  | Baseline weight | 0.995 | 0.971 | 1.018 | <0.0001 |
|  | Treatment D vs A | 0.065 | -0.981 | 1.111 | 0.9027 |
|  | Ethnicity | -0.099 | -1.003 | 0.806 | 0.8309 |
| Dropout | Treatment D vs A | 0.222 | -0.645 | 1.089 | 0.6161 |
|  | Ethnicity | -0.326 | -1.101 | 0.448 | 0.4086 |
| Association parameters | Marker | 0.271 | -1.151 | 1.693 | 0.7086 |
|  | Weight | -0.059 | -0.253 | 0.135 | 0.5504 |

**The bivariate joint model also included data from two dropped arms (B and C) to adjust for informative dropout further and assumed that these patients completed the trial as planned.*

### **Longitudinal outcomes of plasma biomarkers**

Table S23: Model* estimates for longitudinal measurements of adiponectin adjusted for weight changes and informative dropout

| **Component** | **Parameter** | **Estimate** | **95% CI Lower limit** | **95% CI Upper limit** | **P-value** |
| --- | --- | --- | --- | --- | --- |
| Longitudinal Marker | Intercept | 0.555 | 0.297 | 0.812 | <0.0001 |
|  | Time | -0.001 | -0.003 | 0.001 | 0.3345 |
|  | Baseline marker | 0.787 | 0.709 | 0.865 | <0.0001 |
|  | Treatment D vs A | 0.035 | -0.078 | 0.148 | 0.5420 |
|  | Ethnicity | -0.043 | -0.161 | 0.075 | 0.4731 |
| Longitudinal  Weight | Intercept | 0.491 | -1.739 | 2.720 | 0.6661 |
|  | Time | 0.020 | -0.003 | 0.043 | 0.0941 |
|  | Baseline weight | 0.992 | 0.970 | 1.013 | <0.0001 |
|  | Treatment D vs A | 0.084 | -0.932 | 1.100 | 0.8713 |
|  | Ethnicity | -0.054 | -0.931 | 0.824 | 0.9046 |
| Dropout | Treatment D vs A | 0.260 | -0.625 | 1.146 | 0.5644 |
|  | Ethnicity | -0.059 | -0.908 | 0.789 | 0.8912 |
| Association parameters | Marker | 0.415 | -1.394 | 2.223 | 0.6529 |
|  | Weight | -0.034 | -0.242 | 0.174 | 0.7467 |

**The bivariate joint model also included data from two dropped arms (B and C) to adjust for informative dropout further and assumed that these patients completed the trial as planned.*

Table S24: Model* estimates for longitudinal measurements of leptin adjusted for weight changes and informative dropout

| **Component** | **Parameter** | **Estimate** | **95% CI Lower limit** | **95% CI Upper limit** | **P-value** |
| --- | --- | --- | --- | --- | --- |
| Longitudinal Marker | Intercept | 0.893 | 0.419 | 1.367 | 0.0002 |
|  | Time | 0.001 | -0.002 | 0.003 | 0.6639 |
|  | Baseline marker | 0.906 | 0.857 | 0.956 | <0.0001 |
|  | Treatment D vs A | 0.004 | -0.179 | 0.187 | 0.9664 |
|  | Ethnicity | -0.061 | -0.226 | 0.105 | 0.4727 |
| Longitudinal  Weight | Intercept | 1.017 | -1.224 | 3.258 | 0.3738 |
|  | Time | 0.020 | -0.008 | 0.048 | 0.1713 |
|  | Baseline weight | 0.982 | 0.961 | 1.004 | <0.0001 |
|  | Treatment D vs A | 0.148 | -0.817 | 1.113 | 0.7637 |
|  | Ethnicity | 0.155 | -0.682 | 0.991 | 0.7174 |
| Dropout | Treatment D vs A | 0.346 | -0.569 | 1.260 | 0.4588 |
|  | Ethnicity | 0.122 | -0.773 | 1.016 | 0.7893 |
| Association parameters | Marker | 0.050 | -1.425 | 1.524 | 0.9474 |
|  | Weight | -0.108 | -0.433 | 0.218 | 0.5168 |

**The bivariate joint model also included data from two dropped arms (B and C) to adjust for informative dropout further and assumed that these patients completed the trial as planned.*

Table S25: Model* estimates for longitudinal measurements of IL-8 adjusted for weight changes and informative dropout

| **Component** | **Parameter** | **Estimate** | **95% CI Lower limit** | **95% CI Upper limit** | **P-value** |
| --- | --- | --- | --- | --- | --- |
| Longitudinal Marker | Intercept | 1.851 | 1.594 | 2.107 | <0.0001 |
|  | Time | 0.006 | 0.003 | 0.009 | 0.0004 |
|  | Baseline marker | 0.297 | 0.246 | 0.348 | <0.0001 |
|  | Treatment D vs A | 0.041 | -0.111 | 0.193 | 0.5950 |
|  | Ethnicity | 0.103 | -0.042 | 0.249 | 0.1647 |
| Longitudinal  Weight | Intercept | 0.436 | -1.841 | 2.712 | 0.7077 |
|  | Time | 0.020 | -0.002 | 0.043 | 0.0784 |
|  | Baseline weight | 0.991 | 0.969 | 1.013 | <0.0001 |
|  | Treatment D vs A | 0.194 | -0.807 | 1.195 | 0.7042 |
|  | Ethnicity | -0.018 | -0.864 | 0.829 | 0.9675 |
| Dropout | Treatment D vs A | 0.237 | -0.648 | 1.122 | 0.6000 |
|  | Ethnicity | -0.353 | -1.166 | 0.460 | 0.3948 |
| Association parameters | Marker | -0.006 | -1.347 | 1.335 | 0.9927 |
|  | Weight | -0.053 | -0.254 | 0.148 | 0.6050 |

**The bivariate joint model also included data from two dropped arms (B and C) to adjust for informative dropout further and assumed that these patients completed the trial as planned.*

Table S26: Model* estimates for longitudinal measurements of TNF-α adjusted for weight changes and informative dropout

| **Component** | **Parameter** | **Estimate** | **95% CI Lower limit** | **95% CI Upper limit** | **P-value** |
| --- | --- | --- | --- | --- | --- |
| Longitudinal Marker | Intercept | 0.154 | -0.011 | 0.319 | 0.0682 |
|  | Time | 0.007 | 0.004 | 0.009 | <0.0001 |
|  | Baseline marker | 0.642 | 0.561 | 0.723 | <0.0001 |
|  | Treatment D vs A | -0.025 | -0.133 | 0.082 | 0.6412 |
|  | Ethnicity | 0.119 | 0.005 | 0.233 | 0.0415 |
| Longitudinal  Weight | Intercept | 0.433 | -1.940 | 2.806 | 0.7208 |
|  | Time | 0.020 | -0.006 | 0.047 | 0.1374 |
|  | Baseline weight | 0.992 | 0.970 | 1.014 | <0.0001 |
|  | Treatment D vs A | 0.107 | -0.920 | 1.134 | 0.8381 |
|  | Ethnicity | -0.078 | -0.979 | 0.824 | 0.8656 |
| Dropout | Treatment D vs A | 0.425 | -0.495 | 1.345 | 0.3653 |
|  | Ethnicity | -0.236 | -1.052 | 0.579 | 0.5700 |
| Association parameters | Marker | 0.492 | -1.269 | 2.252 | 0.5841 |
|  | Weight | -0.060 | -0.259 | 0.139 | 0.5545 |

**The bivariate joint model also included data from two dropped arms (B and C) to adjust for informative dropout further and assumed that these patients completed the trial as planned.*

Table S27: Model* estimates for longitudinal measurements of resistin adjusted for weight changes and informative dropout

| **Component** | **Parameter** | **Estimate** | **95% CI Lower limit** | **95% CI Upper limit** | **P-value** |
| --- | --- | --- | --- | --- | --- |
| Longitudinal Marker | Intercept | 3.449 | 2.786 | 4.112 | <0.0001 |
|  | Time | 0.004 | 0.002 | 0.005 | <0.0001 |
|  | Baseline marker | 0.591 | 0.518 | 0.665 | <0.0001 |
|  | Treatment D vs A | -0.066 | -0.171 | 0.039 | 0.2201 |
|  | Ethnicity | 0.038 | -0.066 | 0.143 | 0.4699 |
| Longitudinal  Weight | Intercept | 0.265 | -1.993 | 2.522 | 0.8182 |
|  | Time | 0.021 | -0.003 | 0.045 | 0.0877 |
|  | Baseline weight | 0.994 | 0.972 | 1.016 | <0.0001 |
|  | Treatment D vs A | 0.076 | -0.919 | 1.070 | 0.8816 |
|  | Ethnicity | -0.054 | -0.907 | 0.799 | 0.9009 |
| Dropout | Treatment D vs A | 0.263 | -0.608 | 1.134 | 0.5545 |
|  | Ethnicity | -0.192 | -1.075 | 0.691 | 0.6701 |
| Association parameters | Marker | 0.710 | -0.679 | 2.099 | 0.3163 |
|  | Weight | -0.052 | -0.251 | 0.148 | 0.6119 |

**The bivariate joint model also included data from two dropped arms (B and C) to adjust for informative dropout further and assumed that these patients completed the trial as planned.*

Table S28: Model* estimates for longitudinal measurements of hs-CRP adjusted for weight changes and informative dropout

| **Component** | **Parameter** | **Estimate** | **95% CI Lower limit** | **95% CI Upper limit** | **P-value** |
| --- | --- | --- | --- | --- | --- |
| Longitudinal Marker | Intercept | 0.582 | 0.293 | 0.871 | 0.0001 |
|  | Time | 0.001 | -0.003 | 0.006 | 0.6069 |
|  | Baseline marker | 0.612 | 0.527 | 0.697 | <0.0001 |
|  | Treatment D vs A | -0.222 | -0.433 | -0.011 | 0.0388 |
|  | Ethnicity | -0.278 | -0.501 | -0.056 | 0.0141 |
| Longitudinal  Weight | Intercept | 0.378 | -2.039 | 2.795 | 0.7593 |
|  | Time | 0.020 | -0.004 | 0.045 | 0.1074 |
|  | Baseline weight | 0.993 | 0.969 | 1.016 | <0.0001 |
|  | Treatment D vs A | 0.091 | -0.921 | 1.103 | 0.8599 |
|  | Ethnicity | -0.095 | -0.972 | 0.782 | 0.8317 |
| Dropout | Treatment D vs A | 0.321 | -0.579 | 1.222 | 0.4841 |
|  | Ethnicity | -0.213 | -1.014 | 0.588 | 0.6017 |
| Association parameters | Marker | 0.248 | -0.732 | 1.228 | 0.6197 |
|  | Weight | -0.046 | -0.234 | 0.142 | 0.6328 |

**The bivariate joint model also included data from two dropped arms (B and C) to adjust for informative dropout further and assumed that these patients completed the trial as planned.*

###

### **Longitudinal outcomes of urinary biomarkers**

Table S29: Estimates from the final linear mixed effect model for each tertile group for longitudinal NGAL adjusting for two dropped arms (B and C)

|  | **Parameter** | **SE** | **95% CI**  **lower limit** | **95% CI**  **Upper limit** | **p-value** | **Parameter** | **SE** | **95% CI**  **lower limit** | **95% CI**  **Upper limit** | **p-value** | **Parameter** | **SE** | **95% CI**  **lower limit** | **95% CI**  **Upper limit** | **p-value** |
| --- | --- | --- | --- | --- | --- | --- | --- | --- | --- | --- | --- | --- | --- | --- | --- |
| Marker subgroup | **1^st^ Tertile** | | | | | **2^nd^ Tertile** | | | | | **3^rd^ Tertile** | | | | |
| Intercept | 2.271 | 0.469 | 1.345 | 3.197 | <0.0001 | 0.180 | 0.691 | -1.184 | 1.543 | 0.7952 | 1.192 | 0.759 | -0.309 | 2.693 | 0.1186 |
| TIME | 0.005 | 0.004 | -0.003 | 0.013 | 0.2545 | 0.006 | 0.004 | -0.003 | 0.015 | 0.1854 | 0.003 | 0.004 | -0.006 | 0.012 | 0.4670 |
| Baseline NGAL | 0.562 | 0.183 | 0.198 | 0.926 | 0.0028 | 0.480 | 0.227 | 0.031 | 0.930 | 0.0366 | 0.417 | 0.137 | 0.1460 | 0.688 | 0.0030 |
| Age | -0.015 | 0.009 | -0.032 | 0.002 | 0.0873 | 0.011 | 0.010 | -0.008 | 0.030 | 0.2635 | -0.002 | 0.010 | -0.022 | 0.018 | 0.8741 |
| Ethnicity | -0.057 | 0.249 | -0.551 | 0.437 | 0.8194 | 0.094 | 0.275 | -0.452 | 0.640 | 0.7336 | -0.232 | 0.303 | -0.833 | 0.368 | 0.4448 |
| Gender - male | -0.500 | 0.250 | -0.997 | -0.004 | 0.0483 | 0.097 | 0.294 | -0.486 | 0.681 | 0.7413 | 0.048 | 0.307 | -0.562 | 0.657 | 0.8772 |
| Treatment D vs A | -0.215 | 0.207 | -0.627 | 0.196 | 0.3018 | -0.065 | 0.225 | -0.512 | 0.382 | 0.7733 | -0.347 | 0.275 | -0.893 | 0.198 | 0.2093 |

Table S30: Estimates from the final linear mixed effect model for each subgroup for longitudinal ACR* adjusting for two dropped arms (B and C)

|  | **Parameter** | **SE** | **95% CI**  **lower limit** | **95% CI**  **Upper limit** | **p-value** | **Parameter** | **SE** | **95% CI**  **lower limit** | **95% CI**  **Upper limit** | **p-value** |
| --- | --- | --- | --- | --- | --- | --- | --- | --- | --- | --- |
| Marker subgroup | **ACR < 3mg/mmol** | | | | | **ACR >3mg/mmol** | | | | |
| Intercept | 0.336 | 0.668 | -0.999 | 1.672 | 0.6166 | -1.779 | 0.908 | -3.638 | 0.081 | 0.0601 |
| TIME | 0.002 | 0.004 | -0.005 | 0.009 | 0.5727 | 0.007 | 0.007 | -0.006 | 0.021 | 0.2882 |
| Baseline ACR | 0.564 | 0.131 | 0.301 | 0.826 | 0.0001 | 0.877 | 0.221 | 0.406 | 1.347 | 0.0012 |
| Age | -0.007 | 0.013 | -0.032 | 0.018 | 0.5804 | 0.014 | 0.015 | -0.018 | 0.046 | 0.3696 |
| Ethnicity | 0.182 | 0.245 | -0.308 | 0.672 | 0.4616 | 1.159 | 0.403 | 0.300 | 2.019 | 0.0116 |
| Treatment D vs A | -0.074 | 0.253 | -0.578 | 0.431 | 0.7715 | -0.665 | 0.303 | -1.310 | -0.019 | 0.0443 |

**The sample set was divided into two subsets based on KDIGO 2012 clinical practice guideline for the evaluation and management of chronic kidney disease (ACR<3 mg/mmol, [Normal]; ACR> 3mg/mmol,[ microalbuminuria]).*

### **MRI/MRS Sub-study**

Table S31: Summary statistics for internal visceral fat and, intrahepatic and intramyocellular triglyceride content at baseline and 24 weeks by treatment group

|  | **Baseline** | | | | **24weeks** | | | |
| --- | --- | --- | --- | --- | --- | --- | --- | --- |
|  | **Arm A**  **(control)** | **Arm B**  **(20mg)** | **Arm C**  **(40mg)** | **Arm D**  **(80mg)** | **Arm A**  **(control)** | **Arm B**  **(20mg)** | **Arm C**  **(40mg)** | **Arm D**  **(80mg)** |
| Internal visceral fat (dm^3^) | | | | | | | | |
| N | 8 (61.5%) | 7 (70.0%) | 6 (60%) | 8 (80.0%) | 8 (61.5%) | 7 (70%) | 5 (50%) | 8 (80.0%) |
| Mean (SD)  [min. – max.] | 3.5 (1.1)  [1.6 – 5.1] | 5.15 (2.20)  [2.60 – 8.33] | 3.58 (2.70)  [1.78 – 8.79] | 4.10 (2.71)  [0.70 – 8.04] | 3.74 (1.43)  [1.62 – 5.69] | 5.18 (2.67)  [1.93 – 9.55] | 4.42 (2.06)  [2.54 – 7.04] | 4.74 (2.59)  [0.69 - 8.98] |
| Median (IQR) | 3.3 (2.8 – 4.2) | 5.4 (2.8 – 7.2) | 2.3 (2.0 – 4.2) | 3.6 (1.6 – 5.6) | 3.3 (2.9 - 4.7) | 4.9 (2.5 – 7.5) | 3.3 (2.9 – 6.2) | 4.6 (2.4 – 5.8) |
| Missing | 5 (38.5%) | 3 (30.0%) | 4 (40%) | 2 (20.0%) | 5 (38.5%) | 3 (30%) | 5 (50%) | 2 (20.0%) |
| Intrahepatic triglyceride content | | | | | | | | |
| N | 12 (92.3%) | 10 (100.0%) | 8 (80%) | 10 (100.0%) | 8 (62.0%) | 7 (70%) | 4 (40%) | 8 (80.0%) |
| Mean (SD)  [min. – max.] | 8.2 (18.2)  [0.4 – 64.8] | 6.7 (10.4)  [0.4 – 30.7] | 1.9 (2.1)  [0.3 – 6.4] | 2.2 (4.8)  [0.1 – 14.0] | 3.0 (4.1)  [0.1 – 12.8] | 7.3 (10.1)  [0.2 – 25.1] | 1.2 (0.5)  [0.7 – 1.7] | 1.6 (2.7)  [0.3 – 6.9] |
| Median (IQR) | 1.7 (0.6 – 3.0) | 1.6 (0.4 – 8.4) | 1.2 (0.4 – 2.0) | 1.0 (0.6 – 1.5) | 1.8 (0.8 - 2.4) | 2.5 (0.4 – 18.3) | 1.3 (0.7 – 1.5) | 0.6 (0.3 – 1.3) |
| Missing | 1 (7.7%) | 0 (0.0%) | 2 (20%) | 0 (0.0%) | 5 (38.0%) | 3 (30%) | 6 (60%) | 2 (20.0%) |
| Intramyocellular triglyceride content (Soleus) | | | | | | | | |
| N | 12 (92.3%) | 10 (100.0%) | 8 (80%) | 10 (100.0%) | 8 (62.0%) | 7 (70%) | 4 (40%) | 8 (80.0%) |
| Mean (SD)  [min. – max.] | 19.5 (10.8)  [6.2 – 41.8] | 19.0 (17.8)  [6.1 – 65.1] | 12.6 (5.8)  [5.0 – 22.4] | 17.3 (11.8)  [7.4 – 44.5] | 19.5 (13.3)  [5.2 – 40.0] | 17.1 (9.2)  [7.7 – 33.7] | 22.2 (21.0)  [4.8 – 51.9] | 16.6 (9.0)  [8.2 – 33.6] |
| Median (IQR) | 17.8 (11.1 – 23.2) | 12.4 (6.8 - 25.6) | 11.8 (7.2 – 15.2) | 13.7 (9.2 – 21.1) | 16.7 (5.3 - 21.6) | 15.8 (7.8 – 21.5) | 16.1 (4.8 – 21.8) | 14.5 (8.2 – 17.5) |
| Missing | 1 (7.7%) | 0 (0.0%) | 2 (20%) | 0 (0.0%) | 5 (38.0%) | 3 (30%) | 6 (60%) | 2 (20.0%) |
| Intramyocellular triglyceride content (Tibialis anterior) | | | | | | | | |
| N | 11 (84.6%) | 10 (100.0%) | 8 (80%) | 9 (90.0%) | 8 (62.0%) | 7 (70%) | 4 (40%) | 8 (80.0%) |
| Mean (SD)  [min. – max.] | 6.3 (3.1)  [0.0 – 11.0] | 8.1 (3.7)  [1.4 – 13.9] | 5.3 (2.1)  [3.0 – 9.6] | 7.8 (2.8)  [3.0 – 11.0] | 8.1 (5.0)  [2.6 – 18.6] | 10.2 (6.3)  [2.5 – 17.7] | 5.1 (1.4)  [3.8 – 6.6] | 7.4 (2.7)  [3.4 – 11.2] |
| Median (IQR) | 5.9 (4.9 – 9.1) | 8.6 (5.9 – 10.5) | 4.9 (3.6 – 5.4) | 7.7 (7.3 – 9.7) | 7.4 (3.5 - 8.4) | 9.8 (2.8 – 17.6) | 4.9 (3.8 – 5.8) | 7.8 (3.9 – 8.8) |
| Missing | 2 (15.4%) | 0 (0.0%) | 2 (20%) | 1 (10.0%) | 5 (38.0%) | 3 (30%) | 6 (60%) | 2 (20.0%) |

**Table S32: Summary statistics for other MRI/MRS measures (external abdominal fat, total internal fat, total external fat and total body fat) at baseline and 24 weeks by treatment group**

|  | **Baseline** | | | | **24weeks** | | | |
| --- | --- | --- | --- | --- | --- | --- | --- | --- |
|  | **Arm A**  **(control)** | **Arm B**  **(20mg)** | **Arm C**  **(40mg)** | **Arm D**  **(80mg)** | **Arm A**  **(control)** | **Arm B**  **(20mg)** | **Arm C**  **(40mg)** | **Arm D**  **(80mg)** |
| External abdominal fat (dm3) | | | | | | | | |
| N | 8 (61.5%) | 7 (70.0%) | 6 (60%) | 8 (80.0%) | 8 (61.5%) | 7 (70%) | 5 (50%) | 8 (80.0%) |
| Mean (SD)  [min. – max.] | 5.0 (3.6)  [1.7 – 12.2] | 6.4 (3.1)  [2.6 – 10.9] | 4.1 (2.3)  [1.3 – 6.7] | 3.4 (1.8)  [0.8 – 6.7] | 5.1 (3.6)  [1.2 – 12.1] | 6.7 (3.5)  [2.2 – 11.1] | 5.06 (2.3)  [1.8 – 7.5] | 3.8 (2.2)  [0.8 – 8.0] |
| Median (IQR) | 3.8 (1.9 – 6.8) | 6.1 (2.6 – 8.8) | 4.7 (1.5 – 5.9) | 3.2 (2.4 – 3.6) | 3.8 (2.0 - 7.1) | 7.4 (2.4 – 9.1) | 5.0 (4.3 – 6.8) | 3.2 (2.9 – 3.8) |
| Missing | 5 (38.5%) | 3 (30.0%) | 4 (40%) | 2 (20.0%) | 5 (38.5%) | 3 (30%) | 5 (50%) | 2 (20.0%) |
| Total internal fat (dm^3^) | | | | | | | | |
| N | 8 (61.5%) | 7 (70.0%) | 6 (60%) | 8 (80.0%) | 8 (61.5%) | 7 (70%) | 5 (50%) | 8 (80.0%) |
| Mean (SD)  [min. – max.] | 6.1 (1.6)  [3.1 – 7.5] | 8.3 (2.7)  [5.4 – 12.1] | 6.1 (3.7)  [3.6 – 13.4] | 6.8 (4.1)  [2.5 – 12.5] | 6.5 (1.9)  [3.2 – 8.8] | 8.5 (3.2)  [5.5 – 13.9] | 7.1 (2.5)  [4.7 – 9.9] | 7.9 (3.3)  [2.9 – 12.7] |
| Median (IQR) | 6.8 (4.7 – 7.0) | 8.7 (5.8 – 10.8) | 4.7 (3.9 – 6.3) | 6.0 (3.0 – 9.4) | 6.3 (5.0 - 7.9) | 7.5 (5.6 – 11.9) | 6.7 (4.9 – 9.5) | 7.8 (5.0 – 9.3) |
| Missing | 5 (38.5%) | 3 (30.0%) | 4 (40%) | 2 (20.0%) | 5 (38.5%) | 3 (30%) | 5 (50%) | 2 (20.0%) |
| Total external fat (dm^3^) | | | | | | | | |
| N | 8 (61.5%) | 7 (70.0%) | 6 (60%) | 8 (80.0%) | 8 (61.5%) | 7 (70%) | 5 (50%) | 8 (80.0%) |
| Mean (SD)  [min. – max.] | 18.1 (11.0)  [8.1 – 41.1] | 20.4 (7.3)  [9.7 – 30.3] | 13.3 (5.4)  [5.6 – 17.6] | 12.2 (5.4)  [5.0 – 22.8] | 18.3 (11.1)  [6.5 – 40.0] | 20.9 (8.3)  [8.7 – 32.3] | 16.4 (5.4)  [7.6 – 20.9] | 13.3 (5.1)  [5.5 – 22.4] |
| Median (IQR) | 15.0 (9.0 – 20.8) | 22.3 (11.4 – 24.4) | 16.0 (7.2 – 17.5) | 12.0 (7.3 – 12.8) | 14.9 (9.4 – 22.1) | 22.7 (12.1 – 27.8) | 16.9 (16.1 – 20.4) | 12.6 (9.4 – 14.9) |
| Missing | 5 (38.5%) | 3 (30.0%) | 4 (40%) | 2 (20.0%) | 5 (38.5%) | 3 (30%) | 5 (50%) | 2 (20.0%) |
| Total body fat (dm^3^) | | | | | | | | |
| N | 8 (61.5%) | 7 (70.0%) | 6 (60%) | 8 (80.0%) | 8 (61.5%) | 7 (70%) | 5 (50%) | 8 (80.0%) |
| Mean (SD)  [min. – max.] | 24.2 (11.5)  [11.2 – 47.7] | 28.6 (9.5)  [15.5 – 41.2] | 19.4 (7.4)  [9.5 – 29.5] | 19.0 (8.4)  [7.5 – 32.1] | 24.8 (11.6)  [9.6 – 45.9] | 20.4 (11.2)  [14.1 – 44.2] | 23.5 (5.9)  [14.3 – 29.9] | 21.1 (7.6)  [8.1 – 31.7] |
| Median (IQR) | 21.5 (15.9 – 25.5) | 31.0 (16.8 – 36.4) | 20.6 (12.4 – 23.9) | 19.8 (10.4 – 24.3) | 22.2 (16.0 – 27.0) | 30.1 (17.6 – 41.7) | 25.8 (21.7 – 25.9) | 21.5 (15.3 – 25.8) |
| Missing | 5 (38.5%) | 3 (30.0%) | 4 (40%) | 2 (20.0%) | 5 (38.5%) | 3 (30%) | 5 (50%) | 2 (20.0%) |

**Table S33: Model estimates for internal visceral fat (dm3) at 24 weeks**

| **Variable** | **Parameter**  **Estimate** | **Standard**  **Error** | **95% CI** | **p-value** |
| --- | --- | --- | --- | --- |
| Intercept (in dm^3^) | 0.129 | 0.333 | (-0.604, 0.861) | 0.7063 |
| Baseline value of internal visceral fat (in dm^3^) | 1.010 | 0.073 | (0.849, 1.170) | <0.0001 |
| Relative change in total external fat | 4.161 | 1.423 | (1.029, 7.293) | 0.0138 |
| Change in weight (kg) | -0.034 | 0.063 | (-0.173, 0.104) | 0.5941 |
| Treatment D vs control | 0.038 | 0.287 | (-0.593, 0.670) | 0.8961 |

 (n=8 each in Treatment group (Arm D, 80mg) and Control)

**Table S34: Model estimates for intrahepatic triglyceride content at 24 weeks**

| **Variable** | **Parameter**  **Estimate** | **Standard**  **Error** | **95% CI** | **p-value** |
| --- | --- | --- | --- | --- |
| Intercept | 1.359 | 0.375 | (0.542,2.177) | 0.0035 |
| Baseline value of intrahepatic triglyceride content in liver | 0.722 | 0.059 | (0.594,0.850) | <0.0001 |
| Change in weight (kg) | 0.365 | 0.102 | (0.141,0.588) | 0.0039 |
| Treatment D vs control | -1.714 | 0.492 | (-2.787,-0.642) | 0.0045 |

 (n=8 each in Treatment group (Arm D, 80mg) and Control)

Table S35: Model estimates for intramyocellular triglyceride content (soleus and tibialis anterior) at 24 weeks

| **Variable: Soleus** | **Parameter**  **Estimate** | | **Standard**  **Error** | | **95% confidence interval** | | **p-value** | |  |
| --- | --- | --- | --- | --- | --- | --- | --- | --- | --- |
| Intercept | 2.222 | | 3.589 | | (-5.677,10.121) | | 0.5480 | |  |
| Baseline value soleus | 0.902 | | 0.143 | | (0.587,1.216) | | <0.0001 | |  |
| Change in weight (kg) | -0.496 | | 0.724 | | (-2.089,1.097) | | 0.5070 | |  |
| Treatment D vs control | -2.567 | | 2.559 | | (-8.200,3.066) | | 0.3370 | |  |
| **Variable: Tibialis anterior** | |  | |  | |  | |  | |
| Intercept | | 9.882 | | 3.087 | | (3.087,16.678) | | 0.0080 | |
| Baseline value tibialis anterior | | -0.243 | | 0.420 | | (-1.168,0.682) | | 0.5750 | |
| Change in weight (kg) | | -0.060 | | 0.522 | | (-1.089,1.210) | | 0.9100 | |
| Treatment D vs control | | -0.479 | | 2.472 | | (-5.919,4.961) | | 0.8499 | |

  (n=8 each in Treatment group (Arm D, 80mg) and Control)

## **Safety data analysis**

Table S36: Adverse reactions (AR) by severity in each treatment group and in the total patient cohort

| **AR** | **Severity** | **Arm B (20mg)**  **N=84** | | | **Arm C (40mg)**  **N=82** | | | **Arm D (80mg)**  **N=106** | | | **Total randomised**  **N=377** | | |
| --- | --- | --- | --- | --- | --- | --- | --- | --- | --- | --- | --- | --- | --- |
|  |  | **n**  **events** | **n**  **patients** | **%**  **patients** | **n**  **events** | **n**  **patients** | **%**  **patients** | **n**  **events** | **n**  **patients** | **%**  **patients** | **n**  **events** | **n patients** | **% patients** |
| **Abdominal distension** | |  |  |  |  |  |  |  |  |  |  |  |  |
|  | Mild | 0 | 0 | 0.0 | 2 | 2 | 2.4 | 1 | 1 | 0.9 | **3** | **3** | **0.8** |
|  | Moderate | 0 | 0 | 0.0 | 1 | 1 | 1.2 | 0 | 0 | 0.0 | **1** | **1** | **0.3** |
|  | Severe | 0 | 0 | 0.0 | 0 | 0 | 0.0 | 0 | 0 | 0.0 | **0** | **0** | **0.0** |
| **Abdominal pain upper** | |  |  |  |  |  |  |  |  |  |  |  |  |
|  | Mild | 0 | 0 | 0.0 | 0 | 0 | 0.0 | 0 | 0 | 0.0 | **0** | **0** | **0.0** |
|  | Moderate | 0 | 0 | 0.0 | 0 | 0 | 0.0 | 1 | 1 | 0.9 | **1** | **1** | **0.3** |
|  | Severe | 0 | 0 | 0.0 | 0 | 0 | 0.0 | 0 | 0 | 0.0 | **0** | **0** | **0.0** |
| **Acute sinusitis** | |  |  |  |  |  |  |  |  |  |  |  |  |
|  | Mild | 0 | 0 | 0.0 | 1 | 1 | 1.2 | 0 | 0 | 0.0 | **1** | **1** | **0.3** |
|  | Moderate | 0 | 0 | 0.0 | 0 | 0 | 0.0 | 0 | 0 | 0.0 | **0** | **0** | **0.0** |
|  | Severe | 0 | 0 | 0.0 | 0 | 0 | 0.0 | 0 | 0 | 0.0 | **0** | **0** | **0.0** |
| **Ageusia** | |  |  |  |  |  |  |  |  |  |  |  |  |
|  | Mild | 1 | 1 | 1.2 | 0 | 0 | 0.0 | 0 | 0 | 0.0 | **1** | **1** | **0.3** |
|  | Moderate | 0 | 0 | 0.0 | 0 | 0 | 0.0 | 0 | 0 | 0.0 | **0** | **0** | **0.0** |
|  | Severe | 0 | 0 | 0.0 | 0 | 0 | 0.0 | 0 | 0 | 0.0 | **0** | **0** | **0.0** |
| **Amnesia** | |  |  |  |  |  |  |  |  |  |  |  |  |
|  | Mild | 0 | 0 | 0.0 | 0 | 0 | 0.0 | 0 | 0 | 0.0 | **0** | **0** | **0.0** |
|  | Moderate | 0 | 0 | 0.0 | 0 | 0 | 0.0 | 1 | 1 | 0.9 | **1** | **1** | **0.3** |
|  | Severe | 0 | 0 | 0.0 | 0 | 0 | 0.0 | 0 | 0 | 0.0 | **0** | **0** | **0.0** |
| **Angioedema** | |  |  |  |  |  |  |  |  |  |  |  |  |
|  | Mild | 1 | 1 | 1.2 | 0 | 0 | 0.0 | 0 | 0 | 0.0 | **1** | **1** | **0.3** |
|  | Moderate | 0 | 0 | 0.0 | 0 | 0 | 0.0 | 0 | 0 | 0.0 | **0** | **0** | **0.0** |
|  | Severe | 0 | 0 | 0.0 | 0 | 0 | 0.0 | 0 | 0 | 0.0 | **0** | **0** | **0.0** |
| **Anxiety** | |  |  |  |  |  |  |  |  |  |  |  |  |
|  | Mild | 0 | 0 | 0.0 | 1 | 1 | 1.2 | 0 | 0 | 0.0 | **1** | **1** | **0.3** |
|  | Moderate | 0 | 0 | 0.0 | 1 | 1 | 1.2 | 1 | 1 | 0.9 | **2** | **2** | **0.5** |
|  | Severe | 0 | 0 | 0.0 | 1 | 1 | 1.2 | 0 | 0 | 0.0 | **1** | **1** | **0.3** |
| **Arthralgia** | |  |  |  |  |  |  |  |  |  |  |  |  |
|  | Mild | 1 | 1 | 1.2 | 0 | 0 | 0.0 | 0 | 0 | 0.0 | **1** | **1** | **0.3** |
|  | Moderate | 0 | 0 | 0.0 | 1 | 1 | 1.2 | 0 | 0 | 0.0 | **1** | **1** | **0.3** |
|  | Severe | 0 | 0 | 0.0 | 0 | 0 | 0.0 | 0 | 0 | 0.0 | **0** | **0** | **0.0** |
| **Asthenia** | |  |  |  |  |  |  |  |  |  |  |  |  |
|  | Mild | 0 | 0 | 0.0 | 1 | 1 | 1.2 | 0 | 0 | 0.0 | **1** | **1** | **0.3** |
|  | Moderate | 0 | 0 | 0.0 | 0 | 0 | 0.0 | 0 | 0 | 0.0 | **0** | **0** | **0.0** |
|  | Severe | 0 | 0 | 0.0 | 0 | 0 | 0.0 | 0 | 0 | 0.0 | **0** | **0** | **0.0** |
| **Back pain** | |  |  |  |  |  |  |  |  |  |  |  |  |
|  | Mild | 2 | 2 | 2.4 | 0 | 0 | 0.0 | 1 | 1 | 0.9 | **3** | **3** | **0.8** |
|  | Moderate | 0 | 0 | 0.0 | 0 | 0 | 0.0 | 0 | 0 | 0.0 | **0** | **0** | **0.0** |
|  | Severe | 0 | 0 | 0.0 | 0 | 0 | 0.0 | 0 | 0 | 0.0 | **0** | **0** | **0.0** |
| **Burning sensation** | |  |  |  |  |  |  |  |  |  |  |  |  |
|  | Mild | 0 | 0 | 0.0 | 0 | 0 | 0.0 | 0 | 0 | 0.0 | **0** | **0** | **0.0** |
|  | Moderate | 0 | 0 | 0.0 | 1 | 1 | 1.2 | 0 | 0 | 0.0 | **1** | **1** | **0.3** |
|  | Severe | 0 | 0 | 0.0 | 0 | 0 | 0.0 | 0 | 0 | 0.0 | **0** | **0** | **0.0** |
| **Campylobacter gastroenteritis** | | |  |  |  |  |  |  |  |  |  |  |  |
|  | Mild | 0 | 0 | 0.0 | 0 | 0 | 0.0 | 0 | 0 | 0.0 | **0** | **0** | **0.0** |
|  | Moderate | 0 | 0 | 0.0 | 0 | 0 | 0.0 | 1 | 1 | 0.9 | **1** | **1** | **0.3** |
|  | Severe | 0 | 0 | 0.0 | 0 | 0 | 0.0 | 0 | 0 | 0.0 | **0** | **0** | **0.0** |
| **Chest pain** | |  |  |  |  |  |  |  |  |  |  |  |  |
|  | Mild | 0 | 0 | 0.0 | 0 | 0 | 0.0 | 1 | 1 | 0.9 | **1** | **1** | **0.3** |
|  | Moderate | 0 | 0 | 0.0 | 1 | 1 | 1.2 | 0 | 0 | 0.0 | **1** | **1** | **0.3** |
|  | Severe | 0 | 0 | 0.0 | 0 | 0 | 0.0 | 0 | 0 | 0.0 | **0** | **0** | **0.0** |
| **Chromaturia** | |  |  |  |  |  |  |  |  |  |  |  |  |
|  | Mild | 0 | 0 | 0.0 | 0 | 0 | 0.0 | 1 | 1 | 0.9 | **1** | **1** | **0.3** |
|  | Moderate | 0 | 0 | 0.0 | 0 | 0 | 0.0 | 0 | 0 | 0.0 | **0** | **0** | **0.0** |
|  | Severe | 0 | 0 | 0.0 | 0 | 0 | 0.0 | 0 | 0 | 0.0 | **0** | **0** | **0.0** |
| **Confusional state** | |  |  |  |  |  |  |  |  |  |  |  |  |
|  | Mild | 0 | 0 | 0.0 | 0 | 0 | 0.0 | 0 | 0 | 0.0 | **0** | **0** | **0.0** |
|  | Moderate | 0 | 0 | 0.0 | 0 | 0 | 0.0 | 1 | 1 | 0.9 | **1** | **1** | **0.3** |
|  | Severe | 0 | 0 | 0.0 | 0 | 0 | 0.0 | 0 | 0 | 0.0 | **0** | **0** | **0.0** |
| **Constipation** | |  |  |  |  |  |  |  |  |  |  |  |  |
|  | Mild | 0 | 0 | 0.0 | 0 | 0 | 0.0 | 0 | 0 | 0.0 | **0** | **0** | **0.0** |
|  | Moderate | 0 | 0 | 0.0 | 1 | 1 | 1.2 | 0 | 0 | 0.0 | **1** | **1** | **0.3** |
|  | Severe | 0 | 0 | 0.0 | 0 | 0 | 0.0 | 0 | 0 | 0.0 | **0** | **0** | **0.0** |
| **Cough** | |  |  |  |  |  |  |  |  |  |  |  |  |
|  | Mild | 0 | 0 | 0.0 | 1 | 1 | 1.2 | 0 | 0 | 0.0 | **1** | **1** | **0.3** |
|  | Moderate | 0 | 0 | 0.0 | 0 | 0 | 0.0 | 0 | 0 | 0.0 | **0** | **0** | **0.0** |
|  | Severe | 0 | 0 | 0.0 | 0 | 0 | 0.0 | 0 | 0 | 0.0 | **0** | **0** | **0.0** |
| **Depressed mood** | |  |  |  |  |  |  |  |  |  |  |  |  |
|  | Mild | 1 | 1 | 1.2 | 0 | 0 | 0.0 | 0 | 0 | 0.0 | **1** | **1** | **0.3** |
|  | Moderate | 0 | 0 | 0.0 | 0 | 0 | 0.0 | 0 | 0 | 0.0 | **0** | **0** | **0.0** |
|  | Severe | 0 | 0 | 0.0 | 0 | 0 | 0.0 | 0 | 0 | 0.0 | **0** | **0** | **0.0** |
| **Depression** | |  |  |  |  |  |  |  |  |  |  |  |  |
|  | Mild | 0 | 0 | 0.0 | 0 | 0 | 0.0 | 0 | 0 | 0.0 | **0** | **0** | **0.0** |
|  | Moderate | 0 | 0 | 0.0 | 1 | 1 | 1.2 | 0 | 0 | 0.0 | **1** | **1** | **0.3** |
|  | Severe | 0 | 0 | 0.0 | 0 | 0 | 0.0 | 0 | 0 | 0.0 | **0** | **0** | **0.0** |
| **Diarrhoea** | |  |  |  |  |  |  |  |  |  |  |  |  |
|  | Mild | 1 | 1 | 1.2 | 0 | 0 | 0.0 | 6 | 6 | 5.7 | **7** | **7** | **1.9** |
|  | Moderate | 0 | 0 | 0.0 | 3 | 2 | 2.4 | 0 | 0 | 0.0 | **3** | **2** | **0.5** |
|  | Severe | 0 | 0 | 0.0 | 0 | 0 | 0.0 | 0 | 0 | 0.0 | **0** | **0** | **0.0** |
| **Disturbance in attention** | |  |  |  |  |  |  |  |  |  |  |  |  |
|  | Mild | 0 | 0 | 0.0 | 0 | 0 | 0.0 | 0 | 0 | 0.0 | **0** | **0** | **0.0** |
|  | Moderate | 0 | 0 | 0.0 | 0 | 0 | 0.0 | 1 | 1 | 0.9 | **1** | **1** | **0.3** |
|  | Severe | 0 | 0 | 0.0 | 0 | 0 | 0.0 | 0 | 0 | 0.0 | **0** | **0** | **0.0** |
| **Dizziness** | |  |  |  |  |  |  |  |  |  |  |  |  |
|  | Mild | 6 | 6 | 7.1 | 4 | 4 | 4.9 | 13 | 13 | 12.3 | **23** | **23** | **6.1** |
|  | Moderate | 0 | 0 | 0.0 | 2 | 2 | 2.4 | 2 | 2 | 1.9 | **4** | **4** | **1.1** |
|  | Severe | 0 | 0 | 0.0 | 0 | 0 | 0.0 | 1 | 1 | 0.9 | **1** | **1** | **0.3** |
| **Double ureter** | |  |  |  |  |  |  |  |  |  |  |  |  |
|  | Mild | 0 | 0 | 0.0 | 1 | 1 | 1.2 | 0 | 0 | 0.0 | **1** | **1** | **0.3** |
|  | Moderate | 0 | 0 | 0.0 | 0 | 0 | 0.0 | 0 | 0 | 0.0 | **0** | **0** | **0.0** |
|  | Severe | 0 | 0 | 0.0 | 0 | 0 | 0.0 | 0 | 0 | 0.0 | **0** | **0** | **0.0** |
| **Dry eye** | |  |  |  |  |  |  |  |  |  |  |  |  |
|  | Mild | 0 | 0 | 0.0 | 0 | 0 | 0.0 | 2 | 1 | 0.9 | **2** | **1** | **0.3** |
|  | Moderate | 0 | 0 | 0.0 | 0 | 0 | 0.0 | 0 | 0 | 0.0 | **0** | **0** | **0.0** |
|  | Severe | 0 | 0 | 0.0 | 0 | 0 | 0.0 | 0 | 0 | 0.0 | **0** | **0** | **0.0** |
| **Dry mouth** | |  |  |  |  |  |  |  |  |  |  |  |  |
|  | Mild | 0 | 0 | 0.0 | 1 | 1 | 1.2 | 0 | 0 | 0.0 | **1** | **1** | **0.3** |
|  | Moderate | 0 | 0 | 0.0 | 0 | 0 | 0.0 | 0 | 0 | 0.0 | **0** | **0** | **0.0** |
|  | Severe | 0 | 0 | 0.0 | 0 | 0 | 0.0 | 0 | 0 | 0.0 | **0** | **0** | **0.0** |
| **Dry skin** | |  |  |  |  |  |  |  |  |  |  |  |  |
|  | Mild | 0 | 0 | 0.0 | 1 | 1 | 1.2 | 0 | 0 | 0.0 | **1** | **1** | **0.3** |
|  | Moderate | 0 | 0 | 0.0 | 0 | 0 | 0.0 | 0 | 0 | 0.0 | **0** | **0** | **0.0** |
|  | Severe | 0 | 0 | 0.0 | 0 | 0 | 0.0 | 0 | 0 | 0.0 | **0** | **0** | **0.0** |
| **Dysgeusia** | |  |  |  |  |  |  |  |  |  |  |  |  |
|  | Mild | 0 | 0 | 0.0 | 1 | 1 | 1.2 | 0 | 0 | 0.0 | **1** | **1** | **0.3** |
|  | Moderate | 0 | 0 | 0.0 | 0 | 0 | 0.0 | 0 | 0 | 0.0 | **0** | **0** | **0.0** |
|  | Severe | 0 | 0 | 0.0 | 0 | 0 | 0.0 | 0 | 0 | 0.0 | **0** | **0** | **0.0** |
| **Dyspepsia** | |  |  |  |  |  |  |  |  |  |  |  |  |
|  | Mild | 0 | 0 | 0.0 | 0 | 0 | 0.0 | 0 | 0 | 0.0 | **0** | **0** | **0.0** |
|  | Moderate | 2 | 2 | 2.4 | 1 | 1 | 1.2 | 1 | 1 | 0.9 | **4** | **4** | **1.1** |
|  | Severe | 0 | 0 | 0.0 | 0 | 0 | 0.0 | 0 | 0 | 0.0 | **0** | **0** | **0.0** |
| **Ear pain** | |  |  |  |  |  |  |  |  |  |  |  |  |
|  | Mild | 0 | 0 | 0.0 | 1 | 1 | 1.2 | 0 | 0 | 0.0 | **1** | **1** | **0.3** |
|  | Moderate | 0 | 0 | 0.0 | 0 | 0 | 0.0 | 0 | 0 | 0.0 | **0** | **0** | **0.0** |
|  | Severe | 0 | 0 | 0.0 | 0 | 0 | 0.0 | 0 | 0 | 0.0 | **0** | **0** | **0.0** |
| **Ejaculation failure** | |  |  |  |  |  |  |  |  |  |  |  |  |
|  | Mild | 0 | 0 | 0.0 | 0 | 0 | 0.0 | 0 | 0 | 0.0 | **0** | **0** | **0.0** |
|  | Moderate | 0 | 0 | 0.0 | 0 | 0 | 0.0 | 1 | 1 | 0.9 | **1** | **1** | **0.3** |
|  | Severe | 0 | 0 | 0.0 | 0 | 0 | 0.0 | 0 | 0 | 0.0 | **0** | **0** | **0.0** |
| **Epistaxis** | |  |  |  |  |  |  |  |  |  |  |  |  |
|  | Mild | 0 | 0 | 0.0 | 0 | 0 | 0.0 | 2 | 2 | 1.9 | **2** | **2** | **0.5** |
|  | Moderate | 0 | 0 | 0.0 | 0 | 0 | 0.0 | 0 | 0 | 0.0 | **0** | **0** | **0.0** |
|  | Severe | 0 | 0 | 0.0 | 0 | 0 | 0.0 | 0 | 0 | 0.0 | **0** | **0** | **0.0** |
| **Faeces soft** | |  |  |  |  |  |  |  |  |  |  |  |  |
|  | Mild | 0 | 0 | 0.0 | 0 | 0 | 0.0 | 0 | 0 | 0.0 | **0** | **0** | **0.0** |
|  | Moderate | 0 | 0 | 0.0 | 1 | 1 | 1.2 | 0 | 0 | 0.0 | **1** | **1** | **0.3** |
|  | Severe | 0 | 0 | 0.0 | 0 | 0 | 0.0 | 0 | 0 | 0.0 | **0** | **0** | **0.0** |
| **Fall** | |  |  |  |  |  |  |  |  |  |  |  |  |
|  | Mild | 0 | 0 | 0.0 | 0 | 0 | 0.0 | 1 | 1 | 0.9 | **1** | **1** | **0.3** |
|  | Moderate | 0 | 0 | 0.0 | 0 | 0 | 0.0 | 0 | 0 | 0.0 | **0** | **0** | **0.0** |
|  | Severe | 0 | 0 | 0.0 | 0 | 0 | 0.0 | 0 | 0 | 0.0 | **0** | **0** | **0.0** |
| **Fatigue** | |  |  |  |  |  |  |  |  |  |  |  |  |
|  | Mild | 5 | 4 | 4.8 | 3 | 3 | 3.7 | 2 | 2 | 1.9 | **10** | **9** | **2.4** |
|  | Moderate | 0 | 0 | 0.0 | 0 | 0 | 0.0 | 4 | 4 | 3.8 | **4** | **4** | **1.1** |
|  | Severe | 0 | 0 | 0.0 | 1 | 1 | 1.2 | 0 | 0 | 0.0 | **1** | **1** | **0.3** |
| **Feeling cold** | |  |  |  |  |  |  |  |  |  |  |  |  |
|  | Mild | 0 | 0 | 0.0 | 0 | 0 | 0.0 | 1 | 1 | 0.9 | **1** | **1** | **0.3** |
|  | Moderate | 0 | 0 | 0.0 | 0 | 0 | 0.0 | 0 | 0 | 0.0 | **0** | **0** | **0.0** |
|  | Severe | 0 | 0 | 0.0 | 0 | 0 | 0.0 | 0 | 0 | 0.0 | **0** | **0** | **0.0** |
| **Feeling hot** | |  |  |  |  |  |  |  |  |  |  |  |  |
|  | Mild | 0 | 0 | 0.0 | 0 | 0 | 0.0 | 0 | 0 | 0.0 | **0** | **0** | **0.0** |
|  | Moderate | 0 | 0 | 0.0 | 0 | 0 | 0.0 | 1 | 1 | 0.9 | **1** | **1** | **0.3** |
|  | Severe | 0 | 0 | 0.0 | 0 | 0 | 0.0 | 0 | 0 | 0.0 | **0** | **0** | **0.0** |
| **Haematoma** | |  |  |  |  |  |  |  |  |  |  |  |  |
|  | Mild | 0 | 0 | 0.0 | 0 | 0 | 0.0 | 0 | 0 | 0.0 | **0** | **0** | **0.0** |
|  | Moderate | 0 | 0 | 0.0 | 0 | 0 | 0.0 | 1 | 1 | 0.9 | **1** | **1** | **0.3** |
|  | Severe | 0 | 0 | 0.0 | 0 | 0 | 0.0 | 0 | 0 | 0.0 | **0** | **0** | **0.0** |
| **Haematuria** | |  |  |  |  |  |  |  |  |  |  |  |  |
|  | Mild | 0 | 0 | 0.0 | 1 | 1 | 1.2 | 0 | 0 | 0.0 | **1** | **1** | **0.3** |
|  | Moderate | 0 | 0 | 0.0 | 0 | 0 | 0.0 | 0 | 0 | 0.0 | **0** | **0** | **0.0** |
|  | Severe | 0 | 0 | 0.0 | 0 | 0 | 0.0 | 0 | 0 | 0.0 | **0** | **0** | **0.0** |
| **Headache** | |  |  |  |  |  |  |  |  |  |  |  |  |
|  | Mild | 3 | 3 | 3.6 | 5 | 5 | 6.1 | 5 | 5 | 4.7 | **13** | **13** | **3.4** |
|  | Moderate | 2 | 2 | 2.4 | 1 | 1 | 1.2 | 5 | 4 | 3.8 | **8** | **7** | **1.9** |
|  | Severe | 1 | 1 | 1.2 | 1 | 1 | 1.2 | 0 | 0 | 0.0 | **2** | **2** | **0.5** |
| **Hepatic enzyme increased** | | |  |  |  |  |  |  |  |  |  |  |  |
|  | Mild | 0 | 0 | 0.0 | 0 | 0 | 0.0 | 0 | 0 | 0.0 | **0** | **0** | **0.0** |
|  | Moderate | 0 | 0 | 0.0 | 1 | 1 | 1.2 | 0 | 0 | 0.0 | **1** | **1** | **0.3** |
|  | Severe | 0 | 0 | 0.0 | 0 | 0 | 0.0 | 0 | 0 | 0.0 | **0** | **0** | **0.0** |
| **Hyperhidrosis** | |  |  |  |  |  |  |  |  |  |  |  |  |
|  | Mild | 1 | 1 | 1.2 | 1 | 1 | 1.2 | 0 | 0 | 0.0 | **2** | **2** | **0.5** |
|  | Moderate | 1 | 1 | 1.2 | 0 | 0 | 0.0 | 0 | 0 | 0.0 | **1** | **1** | **0.3** |
|  | Severe | 0 | 0 | 0.0 | 0 | 0 | 0.0 | 0 | 0 | 0.0 | **0** | **0** | **0.0** |
| **Hypertension** | |  |  |  |  |  |  |  |  |  |  |  |  |
|  | Mild | 1 | 1 | 1.2 | 0 | 0 | 0.0 | 0 | 0 | 0.0 | **1** | **1** | **0.3** |
|  | Moderate | 0 | 0 | 0.0 | 0 | 0 | 0.0 | 0 | 0 | 0.0 | **0** | **0** | **0.0** |
|  | Severe | 0 | 0 | 0.0 | 0 | 0 | 0.0 | 0 | 0 | 0.0 | **0** | **0** | **0.0** |
| **Hypotension** | |  |  |  |  |  |  |  |  |  |  |  |  |
|  | Mild | 1 | 1 | 1.2 | 2 | 1 | 1.2 | 0 | 0 | 0.0 | **3** | **2** | **0.5** |
|  | Moderate | 0 | 0 | 0.0 | 0 | 0 | 0.0 | 1 | 1 | 0.9 | **1** | **1** | **0.3** |
|  | Severe | 0 | 0 | 0.0 | 0 | 0 | 0.0 | 0 | 0 | 0.0 | **0** | **0** | **0.0** |
| **Increased appetite** | |  |  |  |  |  |  |  |  |  |  |  |  |
|  | Mild | 0 | 0 | 0.0 | 1 | 1 | 1.2 | 0 | 0 | 0.0 | **1** | **1** | **0.3** |
|  | Moderate | 0 | 0 | 0.0 | 0 | 0 | 0.0 | 0 | 0 | 0.0 | **0** | **0** | **0.0** |
|  | Severe | 0 | 0 | 0.0 | 0 | 0 | 0.0 | 0 | 0 | 0.0 | **0** | **0** | **0.0** |
| **Influenza** | |  |  |  |  |  |  |  |  |  |  |  |  |
|  | Mild | 2 | 2 | 2.4 | 1 | 1 | 1.2 | 3 | 3 | 2.8 | **6** | **6** | **1.6** |
|  | Moderate | 0 | 0 | 0.0 | 0 | 0 | 0.0 | 1 | 1 | 0.9 | **1** | **1** | **0.3** |
|  | Severe | 0 | 0 | 0.0 | 0 | 0 | 0.0 | 0 | 0 | 0.0 | **0** | **0** | **0.0** |
| **Influenza like illness** | |  |  |  |  |  |  |  |  |  |  |  |  |
|  | Mild | 0 | 0 | 0.0 | 1 | 1 | 1.2 | 0 | 0 | 0.0 | **1** | **1** | **0.3** |
|  | Moderate | 0 | 0 | 0.0 | 0 | 0 | 0.0 | 0 | 0 | 0.0 | **0** | **0** | **0.0** |
|  | Severe | 0 | 0 | 0.0 | 0 | 0 | 0.0 | 0 | 0 | 0.0 | **0** | **0** | **0.0** |
| **Insomnia** | |  |  |  |  |  |  |  |  |  |  |  |  |
|  | Mild | 1 | 1 | 1.2 | 0 | 0 | 0.0 | 1 | 1 | 0.9 | **2** | **2** | **0.5** |
|  | Moderate | 0 | 0 | 0.0 | 0 | 0 | 0.0 | 2 | 2 | 1.9 | **2** | **2** | **0.5** |
|  | Severe | 0 | 0 | 0.0 | 0 | 0 | 0.0 | 0 | 0 | 0.0 | **0** | **0** | **0.0** |
| **Lacrimation increased** | |  |  |  |  |  |  |  |  |  |  |  |  |
|  | Mild | 0 | 0 | 0.0 | 0 | 0 | 0.0 | 1 | 1 | 0.9 | **1** | **1** | **0.3** |
|  | Moderate | 0 | 0 | 0.0 | 0 | 0 | 0.0 | 0 | 0 | 0.0 | **0** | **0** | **0.0** |
|  | Severe | 0 | 0 | 0.0 | 0 | 0 | 0.0 | 0 | 0 | 0.0 | **0** | **0** | **0.0** |
| **Loss of consciousness** | |  |  |  |  |  |  |  |  |  |  |  |  |
|  | Mild | 0 | 0 | 0.0 | 0 | 0 | 0.0 | 0 | 0 | 0.0 | **0** | **0** | **0.0** |
|  | Moderate | 0 | 0 | 0.0 | 0 | 0 | 0.0 | 1 | 1 | 0.9 | **1** | **1** | **0.3** |
|  | Severe | 0 | 0 | 0.0 | 0 | 0 | 0.0 | 0 | 0 | 0.0 | **0** | **0** | **0.0** |
| **Lower respiratory tract infection** | | |  |  |  |  |  |  |  |  |  |  |  |
|  | Mild | 0 | 0 | 0.0 | 0 | 0 | 0.0 | 0 | 0 | 0.0 | **0** | **0** | **0.0** |
|  | Moderate | 0 | 0 | 0.0 | 0 | 0 | 0.0 | 1 | 1 | 0.9 | **1** | **1** | **0.3** |
|  | Severe | 0 | 0 | 0.0 | 0 | 0 | 0.0 | 0 | 0 | 0.0 | **0** | **0** | **0.0** |
| **Malaise** | |  |  |  |  |  |  |  |  |  |  |  |  |
|  | Mild | 1 | 1 | 1.2 | 0 | 0 | 0.0 | 0 | 0 | 0.0 | **1** | **1** | **0.3** |
|  | Moderate | 0 | 0 | 0.0 | 0 | 0 | 0.0 | 0 | 0 | 0.0 | **0** | **0** | **0.0** |
|  | Severe | 0 | 0 | 0.0 | 0 | 0 | 0.0 | 0 | 0 | 0.0 | **0** | **0** | **0.0** |
| **Morbid thoughts** | |  |  |  |  |  |  |  |  |  |  |  |  |
|  | Mild | 0 | 0 | 0.0 | 0 | 0 | 0.0 | 1 | 1 | 0.9 | **1** | **1** | **0.3** |
|  | Moderate | 0 | 0 | 0.0 | 0 | 0 | 0.0 | 0 | 0 | 0.0 | **0** | **0** | **0.0** |
|  | Severe | 0 | 0 | 0.0 | 0 | 0 | 0.0 | 0 | 0 | 0.0 | **0** | **0** | **0.0** |
| **Mouth ulceration** | |  |  |  |  |  |  |  |  |  |  |  |  |
|  | Mild | 0 | 0 | 0.0 | 0 | 0 | 0.0 | 1 | 1 | 0.9 | **1** | **1** | **0.3** |
|  | Moderate | 0 | 0 | 0.0 | 0 | 0 | 0.0 | 1 | 1 | 0.9 | **1** | **1** | **0.3** |
|  | Severe | 0 | 0 | 0.0 | 0 | 0 | 0.0 | 0 | 0 | 0.0 | **0** | **0** | **0.0** |
| **Myalgia** | |  |  |  |  |  |  |  |  |  |  |  |  |
|  | Mild | 0 | 0 | 0.0 | 0 | 0 | 0.0 | 1 | 1 | 0.9 | **1** | **1** | **0.3** |
|  | Moderate | 0 | 0 | 0.0 | 0 | 0 | 0.0 | 1 | 1 | 0.9 | **1** | **1** | **0.3** |
|  | Severe | 0 | 0 | 0.0 | 0 | 0 | 0.0 | 0 | 0 | 0.0 | **0** | **0** | **0.0** |
| **Nasopharyngitis** | |  |  |  |  |  |  |  |  |  |  |  |  |
|  | Mild | 0 | 0 | 0.0 | 1 | 1 | 1.2 | 1 | 1 | 0.9 | **2** | **2** | **0.5** |
|  | Moderate | 0 | 0 | 0.0 | 0 | 0 | 0.0 | 1 | 1 | 0.9 | **1** | **1** | **0.3** |
|  | Severe | 0 | 0 | 0.0 | 0 | 0 | 0.0 | 0 | 0 | 0.0 | **0** | **0** | **0.0** |
| **Nausea** | |  |  |  |  |  |  |  |  |  |  |  |  |
|  | Mild | 1 | 1 | 1.2 | 3 | 2 | 2.4 | 2 | 1 | 0.9 | **6** | **4** | **1.1** |
|  | Moderate | 0 | 0 | 0.0 | 0 | 0 | 0.0 | 0 | 0 | 0.0 | **0** | **0** | **0.0** |
|  | Severe | 0 | 0 | 0.0 | 0 | 0 | 0.0 | 0 | 0 | 0.0 | **0** | **0** | **0.0** |
| **Neck pain** | |  |  |  |  |  |  |  |  |  |  |  |  |
|  | Mild | 0 | 0 | 0.0 | 0 | 0 | 0.0 | 0 | 0 | 0.0 | **0** | **0** | **0.0** |
|  | Moderate | 1 | 1 | 1.2 | 0 | 0 | 0.0 | 0 | 0 | 0.0 | **1** | **1** | **0.3** |
|  | Severe | 0 | 0 | 0.0 | 0 | 0 | 0.0 | 0 | 0 | 0.0 | **0** | **0** | **0.0** |
| **Neutropenia** | |  |  |  |  |  |  |  |  |  |  |  |  |
|  | Mild | 0 | 0 | 0.0 | 0 | 0 | 0.0 | 1 | 1 | 0.9 | **1** | **1** | **0.3** |
|  | Moderate | 0 | 0 | 0.0 | 0 | 0 | 0.0 | 0 | 0 | 0.0 | **0** | **0** | **0.0** |
|  | Severe | 0 | 0 | 0.0 | 0 | 0 | 0.0 | 0 | 0 | 0.0 | **0** | **0** | **0.0** |
| **Onychomycosis** | |  |  |  |  |  |  |  |  |  |  |  |  |
|  | Mild | 1 | 1 | 1.2 | 0 | 0 | 0.0 | 0 | 0 | 0.0 | **1** | **1** | **0.3** |
|  | Moderate | 0 | 0 | 0.0 | 0 | 0 | 0.0 | 0 | 0 | 0.0 | **0** | **0** | **0.0** |
|  | Severe | 0 | 0 | 0.0 | 0 | 0 | 0.0 | 0 | 0 | 0.0 | **0** | **0** | **0.0** |
| **Oropharyngeal pain** | |  |  |  |  |  |  |  |  |  |  |  |  |
|  | Mild | 0 | 0 | 0.0 | 1 | 1 | 1.2 | 1 | 1 | 0.9 | **2** | **2** | **0.5** |
|  | Moderate | 0 | 0 | 0.0 | 0 | 0 | 0.0 | 0 | 0 | 0.0 | **0** | **0** | **0.0** |
|  | Severe | 0 | 0 | 0.0 | 0 | 0 | 0.0 | 0 | 0 | 0.0 | **0** | **0** | **0.0** |
| **Orthostatic hypotension** | |  |  |  |  |  |  |  |  |  |  |  |  |
|  | Mild | 0 | 0 | 0.0 | 3 | 2 | 2.4 | 1 | 1 | 0.9 | **4** | **3** | **0.8** |
|  | Moderate | 0 | 0 | 0.0 | 0 | 0 | 0.0 | 0 | 0 | 0.0 | **0** | **0** | **0.0** |
|  | Severe | 0 | 0 | 0.0 | 0 | 0 | 0.0 | 0 | 0 | 0.0 | **0** | **0** | **0.0** |
| **Osteopenia** | |  |  |  |  |  |  |  |  |  |  |  |  |
|  | Mild | 0 | 0 | 0.0 | 0 | 0 | 0.0 | 1 | 1 | 0.9 | **1** | **1** | **0.3** |
|  | Moderate | 0 | 0 | 0.0 | 0 | 0 | 0.0 | 0 | 0 | 0.0 | **0** | **0** | **0.0** |
|  | Severe | 0 | 0 | 0.0 | 0 | 0 | 0.0 | 0 | 0 | 0.0 | **0** | **0** | **0.0** |
| **Pain in jaw** | |  |  |  |  |  |  |  |  |  |  |  |  |
|  | Mild | 0 | 0 | 0.0 | 0 | 0 | 0.0 | 1 | 1 | 0.9 | **1** | **1** | **0.3** |
|  | Moderate | 0 | 0 | 0.0 | 0 | 0 | 0.0 | 0 | 0 | 0.0 | **0** | **0** | **0.0** |
|  | Severe | 0 | 0 | 0.0 | 0 | 0 | 0.0 | 0 | 0 | 0.0 | **0** | **0** | **0.0** |
| **Palpitations** | |  |  |  |  |  |  |  |  |  |  |  |  |
|  | Mild | 0 | 0 | 0.0 | 0 | 0 | 0.0 | 2 | 2 | 1.9 | **2** | **2** | **0.5** |
|  | Moderate | 0 | 0 | 0.0 | 0 | 0 | 0.0 | 1 | 1 | 0.9 | **1** | **1** | **0.3** |
|  | Severe | 0 | 0 | 0.0 | 0 | 0 | 0.0 | 0 | 0 | 0.0 | **0** | **0** | **0.0** |
| **Paraesthesia** | |  |  |  |  |  |  |  |  |  |  |  |  |
|  | Mild | 0 | 0 | 0.0 | 0 | 0 | 0.0 | 1 | 1 | 0.9 | **1** | **1** | **0.3** |
|  | Moderate | 0 | 0 | 0.0 | 0 | 0 | 0.0 | 1 | 1 | 0.9 | **1** | **1** | **0.3** |
|  | Severe | 0 | 0 | 0.0 | 0 | 0 | 0.0 | 0 | 0 | 0.0 | **0** | **0** | **0.0** |
| **Photosensitivity reaction** | | |  |  |  |  |  |  |  |  |  |  |  |
|  | Mild | 0 | 0 | 0.0 | 1 | 1 | 1.2 | 0 | 0 | 0.0 | **1** | **1** | **0.3** |
|  | Moderate | 0 | 0 | 0.0 | 0 | 0 | 0.0 | 0 | 0 | 0.0 | **0** | **0** | **0.0** |
|  | Severe | 0 | 0 | 0.0 | 0 | 0 | 0.0 | 0 | 0 | 0.0 | **0** | **0** | **0.0** |
| **Pruritus** | |  |  |  |  |  |  |  |  |  |  |  |  |
|  | Mild | 4 | 4 | 4.8 | 1 | 1 | 1.2 | 2 | 2 | 1.9 | **7** | **7** | **1.9** |
|  | Moderate | 0 | 0 | 0.0 | 0 | 0 | 0.0 | 1 | 1 | 0.9 | **1** | **1** | **0.3** |
|  | Severe | 0 | 0 | 0.0 | 0 | 0 | 0.0 | 0 | 0 | 0.0 | **0** | **0** | **0.0** |
| **Pulmonary fibrosis** | |  |  |  |  |  |  |  |  |  |  |  |  |
|  | Mild | 0 | 0 | 0.0 | 0 | 0 | 0.0 | 0 | 0 | 0.0 | **0** | **0** | **0.0** |
|  | Moderate | 0 | 0 | 0.0 | 0 | 0 | 0.0 | 0 | 0 | 0.0 | **0** | **0** | **0.0** |
|  | Severe | 1 | 1 | 1.2 | 0 | 0 | 0.0 | 0 | 0 | 0.0 | **1** | **1** | **0.3** |
| **Pyrexia** |  |  |  |  |  |  |  |  |  |  |  |  |  |
|  | Mild | 0 | 0 | 0.0 | 0 | 0 | 0.0 | 1 | 1 | 0.9 | **1** | **1** | **0.3** |
|  | Moderate | 0 | 0 | 0.0 | 0 | 0 | 0.0 | 0 | 0 | 0.0 | **0** | **0** | **0.0** |
|  | Severe | 0 | 0 | 0.0 | 0 | 0 | 0.0 | 0 | 0 | 0.0 | **0** | **0** | **0.0** |
| **Rash** | |  |  |  |  |  |  |  |  |  |  |  |  |
|  | Mild | 1 | 1 | 1.2 | 1 | 1 | 1.2 | 2 | 2 | 1.9 | **4** | **4** | **1.1** |
|  | Moderate | 0 | 0 | 0.0 | 0 | 0 | 0.0 | 2 | 2 | 1.9 | **2** | **2** | **0.5** |
|  | Severe | 0 | 0 | 0.0 | 0 | 0 | 0.0 | 0 | 0 | 0.0 | **0** | **0** | **0.0** |
| **Renal impairment** | |  |  |  |  |  |  |  |  |  |  |  |  |
|  | Mild | 0 | 0 | 0.0 | 0 | 0 | 0.0 | 1 | 1 | 0.9 | **1** | **1** | **0.3** |
|  | Moderate | 0 | 0 | 0.0 | 0 | 0 | 0.0 | 0 | 0 | 0.0 | **0** | **0** | **0.0** |
|  | Severe | 0 | 0 | 0.0 | 0 | 0 | 0.0 | 0 | 0 | 0.0 | **0** | **0** | **0.0** |
| **Rhinitis** | |  |  |  |  |  |  |  |  |  |  |  |  |
|  | Mild | 0 | 0 | 0.0 | 0 | 0 | 0.0 | 0 | 0 | 0.0 | **0** | **0** | **0.0** |
|  | Moderate | 0 | 0 | 0.0 | 0 | 0 | 0.0 | 1 | 1 | 0.9 | **1** | **1** | **0.3** |
|  | Severe | 0 | 0 | 0.0 | 0 | 0 | 0.0 | 0 | 0 | 0.0 | **0** | **0** | **0.0** |
| **Sinus congestion** | |  |  |  |  |  |  |  |  |  |  |  |  |
|  | Mild | 1 | 1 | 1.2 | 0 | 0 | 0.0 | 0 | 0 | 0.0 | **1** | **1** | **0.3** |
|  | Moderate | 0 | 0 | 0.0 | 0 | 0 | 0.0 | 0 | 0 | 0.0 | **0** | **0** | **0.0** |
|  | Severe | 0 | 0 | 0.0 | 0 | 0 | 0.0 | 0 | 0 | 0.0 | **0** | **0** | **0.0** |
| **Sinusitis** | |  |  |  |  |  |  |  |  |  |  |  |  |
|  | Mild | 0 | 0 | 0.0 | 0 | 0 | 0.0 | 0 | 0 | 0.0 | **0** | **0** | **0.0** |
|  | Moderate | 0 | 0 | 0.0 | 1 | 1 | 1.2 | 0 | 0 | 0.0 | **1** | **1** | **0.3** |
|  | Severe | 0 | 0 | 0.0 | 0 | 0 | 0.0 | 0 | 0 | 0.0 | **0** | **0** | **0.0** |
| **Somnolence** | |  |  |  |  |  |  |  |  |  |  |  |  |
|  | Mild | 0 | 0 | 0.0 | 1 | 1 | 1.2 | 0 | 0 | 0.0 | **1** | **1** | **0.3** |
|  | Moderate | 0 | 0 | 0.0 | 0 | 0 | 0.0 | 0 | 0 | 0.0 | **0** | **0** | **0.0** |
|  | Severe | 0 | 0 | 0.0 | 0 | 0 | 0.0 | 0 | 0 | 0.0 | **0** | **0** | **0.0** |
| **Syncope** | |  |  |  |  |  |  |  |  |  |  |  |  |
|  | Mild | 1 | 1 | 1.2 | 0 | 0 | 0.0 | 0 | 0 | 0.0 | **1** | **1** | **0.3** |
|  | Moderate | 0 | 0 | 0.0 | 0 | 0 | 0.0 | 0 | 0 | 0.0 | **0** | **0** | **0.0** |
|  | Severe | 0 | 0 | 0.0 | 0 | 0 | 0.0 | 0 | 0 | 0.0 | **0** | **0** | **0.0** |
| **Tension headache** | |  |  |  |  |  |  |  |  |  |  |  |  |
|  | Mild | 0 | 0 | 0.0 | 1 | 1 | 1.2 | 0 | 0 | 0.0 | **1** | **1** | **0.3** |
|  | Moderate | 0 | 0 | 0.0 | 0 | 0 | 0.0 | 0 | 0 | 0.0 | **0** | **0** | **0.0** |
|  | Severe | 0 | 0 | 0.0 | 0 | 0 | 0.0 | 0 | 0 | 0.0 | **0** | **0** | **0.0** |
| **Tongue coated** | |  |  |  |  |  |  |  |  |  |  |  |  |
|  | Mild | 0 | 0 | 0.0 | 0 | 0 | 0.0 | 1 | 1 | 0.9 | **1** | **1** | **0.3** |
|  | Moderate | 0 | 0 | 0.0 | 0 | 0 | 0.0 | 0 | 0 | 0.0 | **0** | **0** | **0.0** |
|  | Severe | 0 | 0 | 0.0 | 0 | 0 | 0.0 | 0 | 0 | 0.0 | **0** | **0** | **0.0** |
| **Tremor** | |  |  |  |  |  |  |  |  |  |  |  |  |
|  | Mild | 0 | 0 | 0.0 | 1 | 1 | 1.2 | 0 | 0 | 0.0 | **1** | **1** | **0.3** |
|  | Moderate | 0 | 0 | 0.0 | 0 | 0 | 0.0 | 0 | 0 | 0.0 | **0** | **0** | **0.0** |
|  | Severe | 0 | 0 | 0.0 | 0 | 0 | 0.0 | 0 | 0 | 0.0 | **0** | **0** | **0.0** |
| **Trigeminal neuralgia** | |  |  |  |  |  |  |  |  |  |  |  |  |
|  | Mild | 1 | 1 | 1.2 | 0 | 0 | 0.0 | 0 | 0 | 0.0 | **1** | **1** | **0.3** |
|  | Moderate | 0 | 0 | 0.0 | 0 | 0 | 0.0 | 0 | 0 | 0.0 | **0** | **0** | **0.0** |
|  | Severe | 0 | 0 | 0.0 | 0 | 0 | 0.0 | 0 | 0 | 0.0 | **0** | **0** | **0.0** |
| **Upper respiratory tract infection** | | |  |  |  |  |  |  |  |  |  |  |  |
|  | Mild | 1 | 1 | 1.2 | 0 | 0 | 0.0 | 0 | 0 | 0.0 | **1** | **1** | **0.3** |
|  | Moderate | 0 | 0 | 0.0 | 0 | 0 | 0.0 | 0 | 0 | 0.0 | **0** | **0** | **0.0** |
|  | Severe | 0 | 0 | 0.0 | 0 | 0 | 0.0 | 0 | 0 | 0.0 | **0** | **0** | **0.0** |
| **Urinary tract infection** | |  |  |  |  |  |  |  |  |  |  |  |  |
|  | Mild | 1 | 1 | 1.2 | 0 | 0 | 0.0 | 0 | 0 | 0.0 | **1** | **1** | **0.3** |
|  | Moderate | 0 | 0 | 0.0 | 0 | 0 | 0.0 | 0 | 0 | 0.0 | **0** | **0** | **0.0** |
|  | Severe | 0 | 0 | 0.0 | 0 | 0 | 0.0 | 0 | 0 | 0.0 | **0** | **0** | **0.0** |
| **Vision blurred** | |  |  |  |  |  |  |  |  |  |  |  |  |
|  | Mild | 1 | 1 | 1.2 | 1 | 1 | 1.2 | 2 | 2 | 1.9 | **4** | **4** | **1.1** |
|  | Moderate | 0 | 0 | 0.0 | 0 | 0 | 0.0 | 0 | 0 | 0.0 | **0** | **0** | **0.0** |
|  | Severe | 0 | 0 | 0.0 | 0 | 0 | 0.0 | 0 | 0 | 0.0 | **0** | **0** | **0.0** |
| **Visual impairment** | |  |  |  |  |  |  |  |  |  |  |  |  |
|  | Mild | 0 | 0 | 0.0 | 0 | 0 | 0.0 | 0 | 0 | 0.0 | **0** | **0** | **0.0** |
|  | Moderate | 1 | 1 | 1.2 | 0 | 0 | 0.0 | 0 | 0 | 0.0 | **1** | **1** | **0.3** |
|  | Severe | 0 | 0 | 0.0 | 0 | 0 | 0.0 | 0 | 0 | 0.0 | **0** | **0** | **0.0** |
| **Vomiting** | |  |  |  |  |  |  |  |  |  |  |  |  |
|  | Mild | 0 | 0 | 0.0 | 1 | 1 | 1.2 | 1 | 1 | 0.9 | **2** | **2** | **0.5** |
|  | Moderate | 0 | 0 | 0.0 | 1 | 1 | 1.2 | 0 | 0 | 0.0 | **1** | **1** | **0.3** |
|  | Severe | 0 | 0 | 0.0 | 0 | 0 | 0.0 | 0 | 0 | 0.0 | **0** | **0** | **0.0** |
| **Weight increased** | |  |  |  |  |  |  |  |  |  |  |  |  |
|  | Mild | 0 | 0 | 0.0 | 1 | 1 | 1.2 | 0 | 0 | 0.0 | **1** | **1** | **0.3** |
|  | Moderate | 0 | 0 | 0.0 | 0 | 0 | 0.0 | 0 | 0 | 0.0 | **0** | **0** | **0.0** |
|  | Severe | 0 | 0 | 0.0 | 0 | 0 | 0.0 | 0 | 0 | 0.0 | **0** | **0** | **0.0** |
| **Grand Total** |  |  |  |  |  |  |  |  |  |  |  |  |  |
|  | **Mild** | **41** | **20** | **23.8** | **47** | **18** | **22.0** | **65** | **27** | **25.5** | **153** | **65** | **17.2** |
|  | **Moderate** | **7** | **6** | **7.1** | **18** | **8** | **9.8** | **36** | **21** | **19.8** | **61** | **35** | **9.3** |
|  | **Severe** | **2** | **2** | **2.4** | **3** | **2** | **2.4** | **1** | **1** | **0.9** | **6** | **5** | **1.3** |

*The numbers of ARs, numbers and % of patients affected in each category by treatment arm by severity are provided in this table. For each patient, only the maximum severity experienced of each type of AR is displayed.*

Table S37: Serious adverse events observed

| **SAE number** | **Description (PT)** | **Description**  **(SOC)** | **Seriousness** | **Allocation** | **Severity** | **Expectedness** | **Relationship**  **PI Assessment** | **Relationship**  **CI Assessment** | **Withdrew from study drug** | **Outcome** | **Patient status** |
| --- | --- | --- | --- | --- | --- | --- | --- | --- | --- | --- | --- |
| 1 | Laceration | Injury, poisoning and procedural complications | Required Hospitalisation | Arm C (40mg) | Grade 4 Potentially life threatening | Unexpected | Unrelated | Unrelated | No | Resolved | Continuing in trial |
| 2 | Plasmablastic lymphoma | Neoplasms benign, malignant and unspecified (incl cysts and polyps) | Medically significant / important  *plasmablastic large B-Cell lymphoma* | Arm C (40mg) | Grade 4 Potentially life threatening | Unexpected | Unrelated | Unrelated | Yes | Ongoing at final follow up | Withdrawn from treatment |
| 3 | Paraesthesia | Nervous system disorders | Required Hospitalisation | Arm C (40mg) | Grade 2 Moderate | Expected | Probably | Probably | Yes | Resolved | Withdrawn from treatment |
| 4 | Mastitis | Infections and infestations | Required Hospitalisation | Arm D (80mg) | Grade 1 Mild | Unexpected | Unrelated | Unrelated | No | Resolved | Continuing in trial |
| 5 | Infected bites | Infections and infestations | Required Hospitalisation | Arm A (control) | Grade 3 Severe | NA | NA | NA | No | Resolved | Continuing in trial |
| 6 | Hepatitis C | Infections and infestations | Medically significant / important  *Hepatitis C* | Arm C (40mg) | Grade 3 Severe | Unexpected | Unlikely | Unrelated | Yes | Resolved | Withdrawn from treatment |
| 7 | Haemoptysis | Respiratory, thoracic and mediastinal disorders | Required Hospitalisation | Arm B (20mg) | Grade 3 Severe | Unexpected | Unrelated | Unrelated | No | Resolved | Continuing in trial |
| 8 | Skin cancer | Neoplasms benign, malignant and unspecified (incl cysts and polyps) | Medically significant / important  *Skin cancer - recurrence* | Arm D (80mg) | Grade 4 Potentially life threatening | Unexpected | Unrelated | Unrelated | No | Not resolved / ongoing | Completed trial |
| 9 | Limb injury | Injury, poisoning and procedural complications | Required Hospitalisation | Arm B (20mg) | Grade 2 Moderate | Unexpected | Unrelated | Unrelated | No | Resolved | Completed trial |
| 10 | Laceration | Injury, poisoning and procedural complications | Required Hospitalisation | Arm A (control) | Grade 1 Mild | NA | NA | NA | No | Resolved | Continuing in trial |
| 11 | Pneumonia | Infections and infestations | Required Hospitalisation | Arm A (control) | Grade 2 Moderate | NA | NA | NA | No | Resolved | Continuing in trial |
| 12 | Joint dislocation | Injury, poisoning and procedural complications | Required Hospitalisation | Arm D (80mg) | Grade 3 Severe | Unexpected | Unlikely | Unrelated | Yes | Resolved | Withdrawn from treatment |
| 13 | Groin pain | Musculoskeletal and connective tissue disorders | Required Hospitalisation | Arm B (20mg) | Grade 3 Severe | Unexpected | Unrelated | Unrelated | No | Ongoing at final follow up | Completed trial |
| 14 | Joint dislocation | Injury, poisoning and procedural complications | Required Hospitalisation | Arm D (80mg) | Grade 3 Severe | Unexpected | Unlikely | Unrelated | Yes | Resolved | Continuing in trial |
| 15 | Convulsion | Nervous system disorders | Required Hospitalisation | Arm A (control) | Grade 2 Moderate | NA | NA | NA | No | Resolved | Continuing in trial |
| 16 | Chest pain | General disorders and administration site conditions | Required Hospitalisation | Arm D (80mg) | Grade 2 Moderate | Unexpected | Unrelated | Unlikely | No | Resolved | Continuing in trial |
| 17 | Death | General disorders and administration site conditions | Required Hospitalisation | Arm A (control) | Grade 5 Death | NA | NA | NA | No | Fatal | Death |
| 18 | Abdominal pain upper | Gastrointestinal disorders | Required Hospitalisation | Arm A (control) | Grade 2 Moderate | NA | NA | NA | No | Resolved | Continuing in trial |
| 19 | Meningitis cryptococcal | Infections and infestations | Required Hospitalisation | Arm D (80mg) | Grade 2 Moderate | Unexpected | Unrelated | Unrelated | No | Resolved | Continuing in trial |
| 20 | Gastroenteritis viral | Infections and infestations | Required Hospitalisation | Arm D (80mg) | Grade 2 Moderate | Unexpected | Unrelated | Unrelated | No | Resolved | Continuing in trial |
| 21 | Pregnancy | Pregnancy, puerperium and perinatal conditions | Medically significant / important  *Pregnancy* | Arm D (80mg) | ** | Unexpected | Unrelated | Unrelated | Yes | Not resolved / ongoing | Completed trial |

*NA - Not applicable as the patient is in the control group*

*** This is a pregnancy related SAE, therefore the severity cannot be completed until the baby is born. The research team have sent in several file notes. The patient moved out of the area and they have been unable to contact her to find out the pregnancy outcome. They have also tried to contact the HIV team at the new hospital, but have received no response. The MHRA were contacted at the time, and was advised that they don’t consider pregnancy to be an SAE.*

## **Compliance with study drug schedule**

Table S38: Patient compliance by treatment arms

|  | **Arm B**  **(20mg)**  **N=84** | **Arm C**  **(40mg)**  **N=82** | **Arm D**  **(80mg)**  **N=106** |
| --- | --- | --- | --- |
| **Total dose (mg) consumed according to the treatment diary**  **Mean (SD), median [min., max.]**  n (%), missing | (n=48)  3215.4 (558.62), 3360 [296, 3360]  36 (42.8) | (n=42)  6347.4 (396.42), 6440 [4042, 6440]  40 (48.8) | (n=64)  11829.8 (893.53),  12040 [5160, 12040]  42 (39.6) |
| **Total dose (mg) according to total number of returned pills**  **Mean (SD), median [min., max.]**  n (%), missing | (n=70)  3290.4 (112.89), 3360 [2880, 3360]  14 (16.7) | (n=61)  6155.4 (413.91), 6440 [4680, 6440]  21 (25.6) | (n=93)  11146.8 (1181.00), 11840 [7620, 12040]  13 (12.3) |
| **Discrepancies between these two estimates of total dose***  **Mean (SD), median [min., max.]**  n (%), missing | (n=43)  52.7 (601.34), 0.0  [-392, 3064]  41 (48.8) | (n=37)  -234.3 (604.41), -120 [-1120, 2398]  45 (54.9) | (n=62)  -964.5 (1534.06), -605.5 [-3857, 6720]  44 (41.5) |
| Average of these two estimates of total dose  Mean (SD), median [min., max.]  n (%), missing | (n=43)  3238.3 (297.51), 3330 [1828, 3360]  41 (48.8) | (n=37)  6217.7 (249.28), 6180 [5241, 6440]  45 (54.9) | (n=62)  11340.8 (745.23), 11512.8 [8520, 12040]  44 (41.5) |

**pill count and treatment diary*

Table S39: Baseline measures according to whether patients provided any compliance data

|  | **No compliance data**  **(n=36)** | **Some compliance data**  **(n=236)** |
| --- | --- | --- |
| **Systolic Blood pressure (mmHg)**  **Mean (SD), median (IQR) [min. - max.]** | 127.5 (13.0), 126.5 (118.5 - 136.5) [105 – 161] | 125.0 (15.0), 122 (115 – 135) [92 – 172] |
| **Diastolic Blood pressure (mmHg)**  **Mean (SD), median (IQR) [min. - max.]** | 76.0 (8.9), 74 (70 – 81) [56 – 100] | 79.2 (11.0), 79 (71 – 87) [54 – 107] |
| **CD4 Cell count (cells/mm3)**  **Mean (SD), median (IQR) [min. - max.]** | 702.4 (255.0), 680 (556 - 846.5) [81 – 1417] | (n=226)  603.1 (261.1), 566.5 (425 – 770) [62 – 1674] |
| **CD4 Cell count & HIV viral load (%)**  **Mean (SD), median (IQR) [min. - max.]** | 31.0 (8.6), 30 (27.5 - 34.5) [6 – 50] | 29.8 (8.5), 30 (24 – 36)  [6 – 52] |
| **HIV viral load - copies/ml)**  **Mean (SD), median (IQR) [min. - max.]** **‡** | (n=13)  16.8 (19.0), 3.5 (0 – 39)  [0 – 39] | (n=69)  40.6 (85.0), 26 (0 – 39)  [0 – 649] |
| **<10, n (%)** | 0 (0.0) | 4 (1.7) |
| **<20, n (%)** | 2 (5.6) | 52 (22.1) |
| **<40, n (%)** | 20 (55.6) | 96 (40.9) |
| **<45, n (%)** | 0 (0.0) | 12 (5.1) |
| **<100, n (%)** | 0 (0.0) | 1 (0.4) |
| **eGFR)**  **Mean (SD), median (IQR) [min. - max.]** **‡** | (n=18)  76.7 (10.6), 77.2 (69 – 85) [53 – 90] | (n=115)  80.5 (12.6), 79 (71 – 87) [56 – 122] |
| **<60, n (%)** | 0 (0.0) | 1 (0.4) |

**References:**

1. Perseghin G, Caumo A, Caloni M, Testolin G, Luzi L. Incorporation of the fasting plasma FFA concentration into QUICKI improves its association with insulin sensitivity in nonobese individuals. J Clin Endocrinol Metab **2001**; 86: 4776-81.

2. Thomas EL, Hamilton G, Patel N, et al. Hepatic triglyceride content and its relation to body adiposity: a magnetic resonance imaging and proton magnetic resonance spectroscopy study. Gut **2005**; 54: 122-7.

3. Magirr D JT, Whitehead J. . A generalized Dunnett test for multi-arm multistage clinical studies with treatment selection. Biometrika **2012**; 99: 494-501.

4. Whitehead J, Jaki T. One- and two-stage design proposals for a phase II trial comparing three active treatments with control using an ordered categorical endpoint. Stat Med **2009**; 28: 828-47.

5. Henderson R, Diggle P, Dobson A. Joint modelling of longitudinal measurements and event time data. Biostatistics **2000**; 1: 465-80.

6. Williamson PR, Kolamunnage-Dona R, Philipson P, Marson AG. Joint modelling of longitudinal and competing risks data. Stat Med **2008**; 27: 6426-38.

7. CRAN Link. Available at: <https://cran.r-project.org/web/packages/joineRML/index.html>. Accessed Accessed on May, 2017.

8. Lin H, McCulloch CE, Mayne ST. Maximum likelihood estimation in the joint analysis of time-to-event and multiple longitudinal variables. Stat Med **2002**; 21: 2369-82.
